# mmu_circ_0012122/mmu-miR-1843-5p/Sertad2 axis: A novel regulatory pathway in rabies virus infection

**DOI:** 10.1080/21505594.2026.2690777

**Published:** 2026-06-17

**Authors:** Qianni Ye, Xinggang Tang, Haiming Cai, Minggui Yuan, Xiaomin Ba, Ya Tian, Jing Chen, Xiaohu Wang, Rong Xiang

**Affiliations:** a Lab of Veterinary Pharmaceutics, Institute of Animal Health, Guangdong Academy of Agricultural Sciences, Guangdong Province Key Laboratory of Livestock Disease Prevention, Guangzhou, P. R. China; b Lab of Parasitology, Institute of Animal Health, Guangdong Academy of Agricultural Sciences, Guangdong Province Key Laboratory of Livestock Disease Prevention, Guangzhou, P. R. China; c Zoonotic & Livestock Diseases (Cattle/Sheep) Research Lab, Institute of Animal Health, Guangdong Academy of Agricultural Sciences, Guangdong Province Key Laboratory of Livestock Disease Prevention, Guangzhou, P. R. China

**Keywords:** Rabies virus, circRNA, expression profile, circRNA-miRNA network

## Abstract

Rabies virus (RABV) causes severe central nervous system damage, though the underlying mechanisms remain unclear. Circular RNAs (circRNAs) have been identified in various cells and tissues and are known to regulate gene expression in eukaryotes. Here, we investigated the expression patterns of circRNAs in brain tissues from mice infected with two strains of RABV (CVS-11 and SRV9) and compared them to brain tissues from uninfected mice. Differential expression analysis identified 1,306 circRNAs, primarily derived from coding exons, with functional enrichment implicating synaptic and nervous system pathways. Critically, we identified and validated a novel regulatory axis, mmu_circ_0012122/mmu-miR-1843-5p/Sertad2, where mmu_circ_0012122 acts as a sponge for mmu-miR-1843-5p, leading to Sertad2 upregulation. This axis differentially modulated murine nerve cell fate: mmu_circ_0012122 knockdown reduced necrotic and non-viable apoptotic cells, while mmu-miR-1843-5p overexpression suppressed viable apoptosis and necrosis. Comprehensive characterization further identified Sertad2 as a key mediator promoting both nerve cell apoptosis and neuroinflammatory responses during infection. These findings underscored circRNAs as critical regulators of neuronal survival during RABV infection and highlighted the mmu_circ_0012122-driven network as a potential therapeutic target.

## Introduction

Circular RNA (circRNA) is a unique molecule resulting from the back splicing of precursor mRNA, characterized by the absence of a 5’end cap or a 3’ end poly(A) tail [1]. It is widely distributed in eukaryotic cells [2]. CircRNAs can be classified into five categories based on their origin: intron-derived, exon-derived, exon-intro-derived, intergenic, and antisense circRNAs. The prediction of circRNAs dates back to 1976 when Sanger et al, used an electron microscope to observe circular structures in viruses [3,4]. In 1991, circRNAs were first discovered in human cells, although initially dismissed as insignificant background noise [5]. In 2013, Hansen et al. proposed the hypothesis that circRNAs function as microRNA sponges and provided the first experimental evidence supporting this notion, which opened up new avenues for circRNA research [6]. The advancement of high-throughput sequencing technology and computational methods for circRNA identification have led to the discovery and extensive analysis of numerous circRNAs [7]. Due to their circular structure and resistance to degradation by exonucleases, circRNAs exhibit greater stability compared to linear RNA molecules. Consequently, circRNAs can interact with precursor miRNAs, competing for splicing sites and influencing parental gene expression and protein synthesis [6, 8–15]. Besides, circular RNAs play a critical role in the development of various diseases based on comprehensive mechanisms and may be useful as biomarkers and potential targets for the diagnosis and treatment of diseases in clinic practice [16–20]. Currently, the mechanism and function of circRNAs have attracted more and more attention, making it a hotspot in molecular biology [21]. Recently, research on circRNAs and their role as microRNA sponges in farmed animals has primarily focused on aspects such as the growth and development, fat deposition, and reproductive features of animals [22–24]. However, the function of circRNAs in the models of viral illness has not been fully documented. In humans, Ghosal et al. discovered that 144 viral miRNAs potentially targeted 1,277 human circRNA transcripts, forming a competitive endogenous RNA (ceRNA) network that facilitates host-virus interaction [25]. These circRNAs exhibited numerous miRNA-binding sites, suggesting their involvement as microRNA sponges, thereby modulating viral infection outcomes and associated processes.

Rabies virus (RABV) is a single-stranded negative-sense RNA virus belonging to the Lyssavirus genus of the family Rhabdoviridae, with attenuated strains like SRV9 and virulent strains like CVS-11 triggering distinct host responses. It can infect a wide range of warm-blooded animals, causing a pronounced neutrophilia [26, 27]. Almost all people infected with RABV will eventually die, and no effective treatment has been discovered up to date. To elucidate the pathogenic mechanism of RABV, it is crucial to investigate the cellular and molecular connections between the host and virus. Novel technologies, such as transcriptome and proteome, have been widely used to study the infection and pathogenesis of RABV at various levels, including DNA/mRNA, miRNA, and protein dynamics [28–30]. Despite these advancements, the role of circRNAs in the host-virus interaction network during RABV infection remains largely unknown, and studies on the mechanism of circRNA in RABV infection and regulation have been less reported. Here, we employed RNA sequencing of RABV-infected mouse brains to identify differentially expressed circRNAs (DECs) and constructed a novel ceRNA network centered on mmu_circ_0012122. We further validated its interaction with mmu-miR-1843-5p and downstream regulation of Sertad2, revealing strain-specific effects on neuronal viability. This study established the direct link between circRNA-mediated networks and RABV neuropathogenesis, offering new insights into host-virus interactions.

## Materials and methods

### Ethics standards

During all the procedures, all efforts were made to minimize animal suffering. This study was approved by the Animal Care Committee of Institute of Animal Health, Guangdong Academy of Agricultural Sciences in China (Experimental Animal Use License Number: SYXK(粤) 2021–0165; Experimental Animal Ethics Approval Number: SPF2017028).

### Animals, cells and viruses

A total of 27 specific pathogen-free (SPF) Kunming suckling mice (3-day-old) were purchased from the Guangdong Laboratory Animals Monitoring Institute and maintained in controlled environmental conditions with free access to water and food. Two strains of the rabies virus (CVS-11 and SRV9) were used in this study to investigate the host response to strains with different degrees of virulence. These two strains were gifts from the laboratory of Researcher Rongliang Hu, Institute of Military Veterinary Medicine, Academy of Military Medical Sciences and stored in our laboratory. The CVS-11 strain, which is highly pathogenic, was chosen to represent a highly virulent strain. This strain has been extensively studied and is known for its ability to cause severe symptoms in infected animals. Additionally, the SRV9 strain was utilized as an attenuated virus derived from the SAD strain through plaque purification in baby hamster kidney (BHK) cells. This strain was selected to represent a less virulent or attenuated strain. The SRV9 strain has undergone genetic modifications to reduce its virulence, making it a valuable tool for studying the host immune response to RABV infection. The viruses were grown in N2A cells (derived from A/J mouse) using Dulbecco’s modified Eagle’s medium (DMEM; Gibco, CA, USA) supplemented with 2% fetal bovine serum (FBS; Gibco, CA, USA). After titration, the virus stocks were aliquoted and stored at −80 °C until further use.

### RABV infections

The mice were randomly divided into three groups after one week of quarantine, with three replicates per group and three mice per replicate. Group C was infected with the RABV strain CVS-11, Group S was infected with the RABV strain SRV9, and Group N was inoculated with a suspension of normal mouse brain tissue homogenized in DMEM. This suspension was prepared using the brain tissue of healthy SPF Kunming suckling mice purchased from the Guangdong Laboratory Animals Monitoring Institute and homogenized in DMEM under sterile conditions as a control to mimic the mechanical effects of intracranial injection without introducing viral particles. A 30 μl suspension containing 10^5^ fluorescent focus units (FFU) of either CVS-11 or SRV9 was intracranially injected into the head of the mice using a microsyringe. The injection point was determined as the vertex of an equilateral triangle with the line connecting the two eyes of the mouse as the base, and the suspension was vertically injected at this point. After injection, the mice were marked and returned to their cages. All procedures were performed in a biosafety cabinet.

Each mouse was sacrificed by an intraperitoneal injection of a high dose of ketamine (300 mg/kg) and xylazine (30 mg/kg). Death was confirmed by the absence of respiration and heartbeat. Subsequently, 75% alcohol was used for disinfection, and then autoclaved scissors and tweezers were used to peel the skin of the top of the head and neck of the mouse, exposing the skull. The skull was fixed with tweezers, and the marginal area at the base of the skull was cut with scissors. The skull was lifted to expose the brain tissue, and then the intact brain tissue was carefully removed. A total of 27 brain tissues were collected finally.

### RNA preparation and quantitation

Total RNA extraction was performed by mirVanaTM miRNA Isolation Kit (Ambion, catalog number: AM1561). RNA quality was assessed by electrophoresis using Agilent Bioanalyzer 2100 (Agilent technologies, Santa Clara, CA, USA). Purification was performed using RNAClean XP Kit (Beckman Coulter, catalog number: A63987) and Rnase-Free Dnase Set (QIAGEN, catalog number: 74,204). RNA quality was re-assessed using NanoDrop ND-2000 spectrophotometer (Thermo Fisher Scientific, MA, USA) and Agilent Bioanalyzer 2100, and the qualified RNA was reserved for subsequent experiments. A total of 27 samples were obtained and mixed before sequencing, with the 3 mice per replicate in a group mixed into one. Finally, 3 mixed samples of RNAs per group were formed. There were 9 samples in total for RNA sequencing in this study.

### circRNAs library preparation and sequencing

The total RNA was subjected to rRNA removal using Ribo-Zero rRNA Removal Reagents (human/mouse/rat) (Illumina, catalog number: RZG1224), followed by RNase R digestion (Epicenter, catalog number: RNR07250) for circular RNA enrichment. Library preparation was performed using the VAHTS Total RNA sequencing (H/M/R) Library Prep Kit for Illumina (Vazyme, catalog number: NR603-02), which included fragmentation, cDNA synthesis, terminal repair, 3’ terminal A addition, adapter ligation and PCR enrichment. Agencourt AMPure XP Beads (Beckman, catalog number: A63881) were used for purification steps throughout the library preparation process. RNA concentration in the constructed library was measured using the Qubit™ dsDNA HS Assay Kit with a Qubit® 2.0 Fluorometer (Invitrogen, Carlsbad, CA, USA), and RNA integrity was evaluated using the Agilent High Sensitivity DNA Kit (Agilent, catalog number: 5067–4626) on an Agilent Bioanalyzer 2100. The libraries were sequenced using an Illumina HiSeq 2500 with a Paired-End module. All library preparation and sequencing steps were performed by Shanghai Biotechnology Corporation (Shanghai, China).

### circRNA prediction and annotation

The reads were filtered by Seqtk version 1.2 [31] and those with adapters, low quality (Q < 20), too short reads (read length < 25) or ribosome RNA reads were removed [31]. Clean reads were then mapped to reference genome (GRCm38.p4, mm10, ftp://ftp.ensembl.org/pub/release-83/fasta/mus_musculus/dna/Mus_musculus.GRCm38.dna.primary_assembly.fa.gz) with default parameters in BWA-MEM version 0.7.8 [32]. CircRNAs were predicted by CIRI [33] and annotated by circBase [34] to discriminate the known and novel circRNAs.

### Differential expression analysis

Gene expression was calculated based on junction reads at circRNA back-splicing sites [35], and normalized by using the circRNA spliced reads per billion mapped reads (SRPBM). Besides, R package edgeR package version 3.12.1 [34] was used to carry out differential expression analysis of circRNAs. After the *p*-value obtained, the correction of multiple hypothesis testing was performed and the threshold value of *p*-value was determined by adjusting false discovery rate (FDR) [36, 37]. The corrected *p*-value was the q-value. Meanwhile, we estimated the folds of differential expression, named fold change, based on the SRPBM value. The differently expressed genes were identified according to the criteria of *p*-value < 0.05 and log_2_(fold change)>1 or log_2_(fold change)<−1 [38, 39]. Specifically, log_2_(fold change) is inf and – inf respectively indicating that the expression level is upregulated from 0 and downregulated to 0, respectively [40].

### GO and KEGG enrichment of parental genes of circRNAs

The corresponding parental genes were retrieved according to the genomic location of circRNAs. The parental genes of the differently expressed circRNAs were used for Gene Ontology (GO) analysis. The number of genes classified into biological process (BP), cellular component (CC) and molecular function (MF) were counted, respectively, using R package org.Mm.eg.db version 3.16.0 for gene annotation. In addition, we performed GO enrichment analysis using R package clusterProfiler version 4.6.2. GO terms with q-value ≤ 0.05 were defined as significantly enriched ones in differentially expressed genes (DEGs). Results of GO enrichment analysis was presented with as a scatter plot using R package ggplot2 version 3.4.4. Gene Ratio refers to the ratio of the number of the parental genes of DECs enriched in the GO term to the total number of genes in this GO terms. Similar with GO analysis, parental genes of DECs in each pathway were analyzed and KEGG pathway enrichment analysis was performed using R package clusterProfiler. A bigger Gene Ratio and a smaller q-value suggested a more significant enrichment of pathways. All analyses were done using RStudio software version 2022.07.2 build 576 and R software version 4.2.2.

### Targeted miRNA prediction of circRNAs and construction of interaction network

Based on the circRNA-related genes, Targetscan (release 7.1, http://www.targetscan.org/) and miRnada (version 3.3a, http://www.microrna.org/microrna/home.do) were used to predict the target miRNAs of DECs. The miRNAs were predicted for all DECs, and the interaction network between the DECs and the predicted target miRNAs was constructed using Cytoscape version 3.8.2.

### Validation of circular structure of mmu_circ_0012122

After assessment, mmu_circ_0012122 was screened out and validated for the circular structure using PCR with divergent and convergent primers, preparing for further study. The RNA and gDNA of the brain tissues of mice were extracted, respectively. The RNA was divided into two parts, of which one was reversely transcribed, and the other was digested by Rnase R enzyme (Abcam, Cat.No. ab286929) to eliminate linear RNA and then reversely transcribed. Divergent primers were designed in regions about 100bp from a junction, and convergent primers were designed of one exon (Details of primers were provided in Table S3). To confirm the junction sequences of circRNAs, PCR products of divergent primers were gel purified and submitted for Sanger sequencing at Sangon Biotech (Shanghai, China).

### Cell transient transfection

To explore whether the three miRNAs with predictive binding sites, namely mmu-miR-139-3p, mmu-miR-1843-5p and mmu-miR-193a-5p, interact with mmu_circ_001212, the 812bp sequence in the corresponding binding site was selected and the wild-type and mutant sequences were separately designed and constructed, and then the fragments were ligated into the psiCHECK2 vector using XhoI and NotI enzymes (Table S4).

One day prior to transfection, 5 × 10^6^ N2A cells were seeded in 10 cm dishes with 9 mL DMEM complete medium and incubated at 37°C/5% CO_2_ to reach 70–90% confluency. For transfection, 12 μg plasmid DNA and 36 μL Lipofectamine 2000 (Invitrogen, Cat.No.11668019) were separately diluted in 500 μL serum-free medium each. After 5 min incubation, the solutions were combined, mixed gently, and incubated for 15 min at room temperature to form complexes. The 1 mL complex mixture was added dropwise to cells, followed by gentle rocking. Post-24 h incubation (37°C/5% CO_2_), medium was replaced with fresh complete medium containing 0.5 μg/mL Doxycycline Hyclate (Selleck, Cat.No. S4163) to induce protein capture. Samples were harvested after 48 h induction for fluorescence imaging analysis.

### Pull-down assay

The transfected N2A cells were lysed in 500 μL buffer with protease/RNase inhibitors, followed by 3–5 liquid nitrogen freeze-thaw cycles. After centrifugation, supernatants were stored at −80°C. For immunoprecipitation, Protein A/G magnetic beads (Bimake, Cat.No. B23201) were washed in binding buffer, conjugated with 5 μg antibody at 4°C for 1–2 h, then incubated with 400 μL lysate in binding buffer for 2 h. Post-incubation, beads were stringently washed. Complexes were eluted using 300 μL Buffer E (Inputs: 200 μL). Eluates underwent DNA removal via DR Columns (14,000×g, 2 min), then mixed with equal-volume 70% ethanol and loaded onto RC Columns. After centrifugation (12,000×g, 30–60 s), protein-containing flow-through was collected. RNA was purified on RC Columns through sequential washes with Buffer F (500 μL) and Buffer G (2 × 500 μL), followed by ethanol removal (12,000×g, 2 min) and elution in RNase-free water (30–100 μL, 2 min incubation; 12,000×g, 1 min). RNA was stored at −80°C. Protein was precipitated from the flow-through using 4× volume ice-cold acetone, washed with ethanol, air-dried, and resuspended in appropriate buffers for Western blotting. Anti-Flag antibodies (Genscript, Cat.No. A00170) and goat Anti-Mouse IgG Antibody (Genscript, Cat.No. A01008) were used. RNA was extracted for RT-qPCR analysis to detect circRNA enrichment.

### Dual-luciferase reporter assays

For the dual-luciferase reporter assays, all procedures were performed following strict standardized protocols. Briefly, one night prior to transfection, 1 × 10^5^ 293T cells were seeded into 24-well plates to ensure 70–80% confluency at the time of transfection. For co-transfection, recombinant psiCHECK2 plasmids (1 μg in 50 μL serum-free medium) were co-transfected into cells using 2 μL Lipofectamine 2000 (Invitrogen, Cat.No. 31,985,070) diluted in another 50 μL serum-free medium according to the manufacturer’s instructions. After 6 h of incubation at 37°C with 5% CO_2_, the transfection medium was removed and replaced with fresh complete medium. After 48 h of transfection, cells were lysed and the activities of firefly and renilla luciferase were detected with a dual-luciferase assay kit. The relative fluorescence activity was calculated as the activity ratio (Renilla luciferase/Firefly luciferase, R/F).

### Cell proliferation and apoptosis detection

N2A cells transfected with control, si-circ_0012122, or mmu-miR-1843-5p mimics were analyzed for proliferation and apoptosis. For proliferation assays, cells were plated in 96-well plates and quantified using CCK-8 Kit (YEASEN, Cat.No. 40203ES60) with OD_450 nm_ measurement according to manufacturer’s protocol. Apoptosis was evaluated via flow cytometry using Annexin V-FITC/PI Cell Apoptosis Detection Kit (YEASEN, Cat.No. 40302ES60) according to manufacturer’s protocol.

### TUNEL staining

For TUNEL staining, N2A cells were cultured on Lab-Tek Chamber Slides and subjected to the corresponding transfection and RABV infection treatments. After treatment, N2A cells were stained using the TUNEL staining kit (KeyGEN BioTECH, Cat.No. KGA1405-100) according to the manufacturer’s instructions. Briefly, cells were gently washed twice with PBS, fixed with 4% paraformaldehyde at room temperature for 20 min, and rinsed three times with PBS for 5 min each. Cells were then permeabilized at room temperature for 5 min, washed repeatedly in PBS, and treated with 3% H_2_O_2_ (prepared in PBS) at room temperature for 20 min, followed by three additional PBS washes. After equilibration, cells were incubated with TdT incubation buffer in the dark, followed by staining with Streptavidin-TRITC and DAPI nuclear counterstaining. Finally, slides were mounted with anti-fade medium, and TUNEL-positive cells were observed and quantified under a fluorescence microscope for statistical analysis.

### Data analysis and processing

Multiple comparisons were performed using one-way and two-way analyses of variance (ANOVA), followed by a Tukey’s post hoc test, whereas two-group comparisons were conducted using two-tailed Student’s t-test. All results are presented as mean ± s.d., and differences for which *p* < 0.05 were considered significant. Significance levels were defined as follows: n.s. (not significant, *p* > 0.05), *p < 0.05, **p < 0.01, ***p < 0.001 and ****p < 0.0001.*p* values were analyzed using GraphPad Prism 8 software (GraphPad Software) and are indicated in the figures.

## Results

### Validation of RABV infection in mouse brain

In order to analyze the changes of circRNAs expression induced by RABV infection, we infected mice with RABV strain SRV9 (Group S) or CVS-11 (Group C) and used suspension from normal brain tissue as a control group (Group N). Daily observations were made for all groups. Mice infected with the SRV9 strain showed symptoms such as trembling, weak hindlimb, and depression, whereas those infected with the CVS-11 strain had ruffled fur, hindlimb paralysis, and poor mental state, which were apparently attributed to virulence differences (Figure S1). Following euthanasia, brain samples were collected from mice in all 3 groups. The viral titers were examined by the Reed-Muench method and results showed that they were 10^5^ TCID_50_/mL and 10^4^ TCID_50_/mL in the SRV9 and CVS-11 strains, confirming the RABV infection in mouse brain.

### Characteristics of circRNAs expressed in mouse brain by RNA sequencing

**Figure 1.**
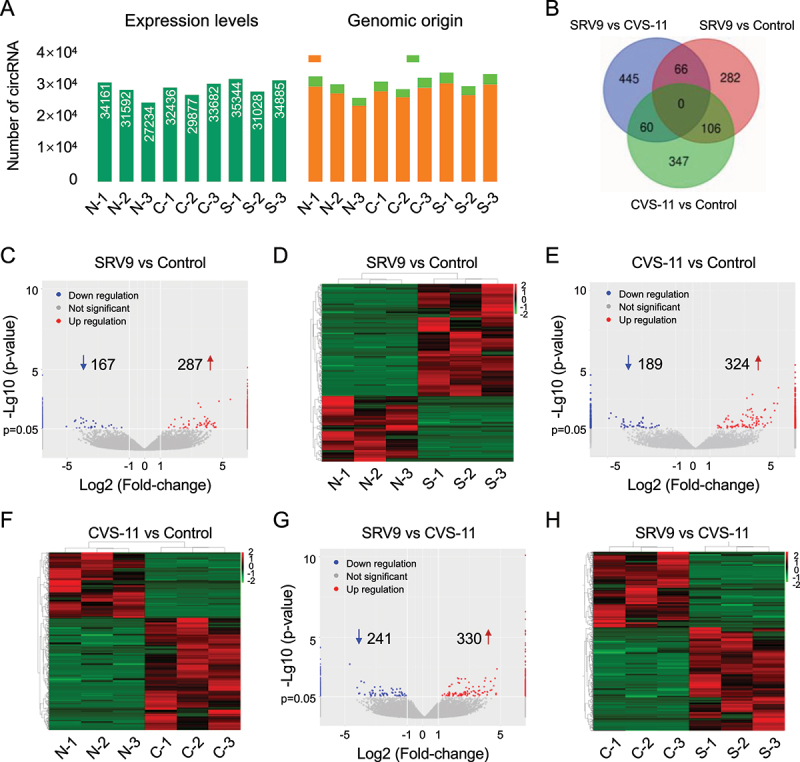
Differential expression pattern of circRNAs in mouse brain infected with rabies virus. (A) Bar plot illustrating the expression levels of circRNAs and the numbers of circRNAs derived from exonic and intronic regions in group N (control mice), group S (SRV9 infected mice), or group C (CVS-11 infected mice). (B) Venn diagram showing the overlap among three different comparison groups. (C), (E), (G) volcano plots were generated to visualize the differential expression of circRNAs in the following comparisons: SRV9 vs control, CVS-11 vs control, and SRV9 vs CVS-11. The volcano plots were constructed using log2 (fold-change) and *p*-value. Up-regulated circRNAs are represented by red points, down-regulated circRNAs are represented by blue points, and circRNAs with no significant difference are represented by gray points. The cutoff criteria were set as log2 (fold-change) >2, log2 (fold-change) <−2 and *p*-value < 0.05. (D), (F), (H) hierarchical clustering analysis was performed to investigate the expression patterns of differentially expressed circRNAs across the comparisons of SRV9 vs control, CVS-11 vs control, and SRV9 vs CVS-11. The expression levels were represented using different colors, where a red strip indicated high relative expression and a green strip indicated low relative expression.

In the RNA sequencing analysis [41], a total of approximately 750 million reads were generated from all nine samples, with each sample yielding about 80 million reads. The raw data were processed to remove adapter and low quality sequences, and then mapped to GRCm38.p4 (mm10) using BWA-MEM with default parameters. With the exception of chromosome Y, the mapping reads were spread out evenly over the chromosome (Figure S2). There were abundant circRNAs expressed in the brain tissues of mice. Among the nine libraries, the proportion of clean reads mapped to the GRCm38.p4 (mm10) genome ranged from 99.48% to 99.72% (Table S1). The average number of circRNAs was 30,804 ± 2,504 (Figure 1A). The average number of circRNAs in Group N was determined to be 29,574 ± 3,354, while Group C had an average of 30,613 ± 1,792 circRNAs, and Group S had an average of 32,224 ± 2,298 circRNAs. These results indicate that RABV infection leads to a higher abundance of circRNAs in mouse brain tissues. To further analyze the characteristics of circRNAs, exonic, intronic, and intergenic circRNAs were distinguished and statistically analyzed using perl scripts. Among these, 90.40% of circRNAs were derived from exons, while 9.60% were derived from intronic regions (Figure 1A). A total of 10,737 genes were found to produce circRNAs, with varying circRNA production levels per gene. Majority of the genes (74.07%, 7,953/10,737) produced 1 to 10 circRNAs, while 2,784 genes generated more than 10 circRNAs. Furthermore, 1,256 out of the 10,737 genes (11.7%) produced more than 20 circRNAs. Notably, certain genes such as small_nucleolar_RNA_host_gene_14 (Snhg14), neurobeachin (Nbea), and CUB_and_Sushi_multiple_domains_1(Csmd1) were capable of producing up to 100 circRNAs (Figure S3).

### Expression analysis of circRNAs

The circRNAs expressed in the brains of mice infected with virulent strain CVS-11 (Group C) and attenuated strain SRV9 (Group S) were compared to those of the control group (Group N). Sequencing results revealed 1,306 DECs in three different comparison groups, with Venn diagram showing the overlap (Figure 1B). Specifically, A total of 454 DECs were identified between Group S and Group N. Compared to Group N, Group S had 287 up-regulated circRNAs and 167 down-regulated circRNAs expression (Figure 1C,D). A total of 513 DECs were identified between Group C and Group N. Compared to Group N, Group C had 324 up-regulated circRNAs and 189 down-regulated circRNAs expression (Figure 1E,F). Moreover, a total of 571 DECs were identified between Group S and Group C. Compared to Group C, Group S had 330 up-regulated circRNAs and 241 down-regulated circRNAs expression (Figure 1G,H). These findings suggest that RABV infection significantly disrupts the host circRNA expression profile. The greater number of DECs in Group C vs Group N compared to Group S vs Group N may reflect the more severe pathological impact of the virulent strain. Furthermore, the substantial differences between Group S and Group C highlight potential circRNA involvement in modulating viral pathogenicity and host immune response. Future functional studies on these DECs could elucidate their roles in RABV pathogenesis and uncover novel therapeutic targets.

### GO enrichment of circRNAs’ parental genes

**Figure 2.**
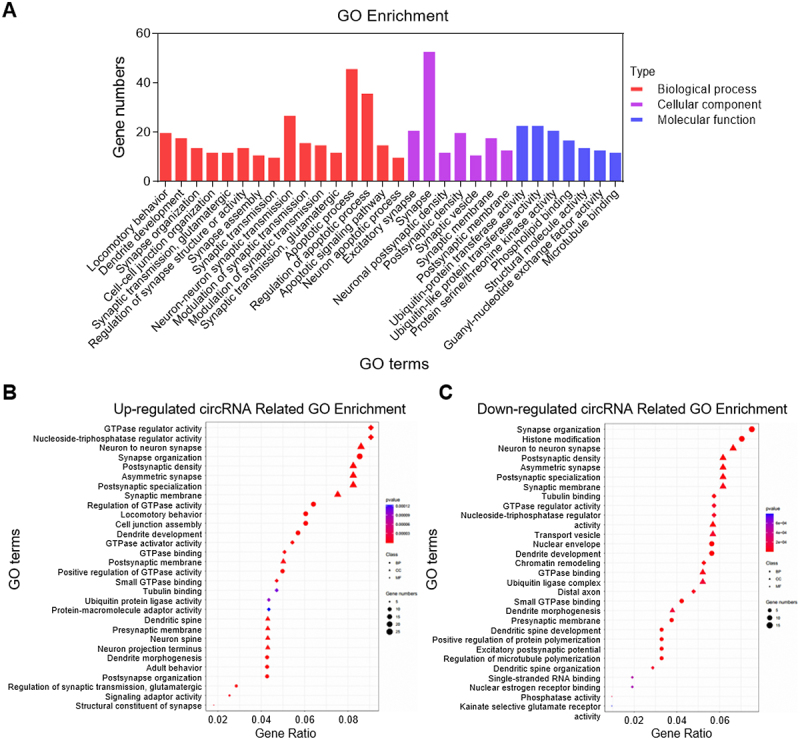
GO enrichment analysis of differentially expressed circRNAs related parental genes in mouse brain infected with attenuated strain SRV9 compared with virulent strain CVS-11. (A) Bar plots illustrate the representation of GO terms in group S vs group C. The Y-axis represents the number of circRNA-related parental genes enriched in GO terms, while the X-axis represents the enriched GO terms. Different colors indicate biological processes, cellular components, and molecular functions. Bubble plots display the top 10 enriched GO terms that were up-regulated (B) or down-regulated (C) for the differentially expressed circRNAs related to parental genes in group S vs group C. The Y-axis represents the GO terms, and the X-axis represents the gene ratio of circRNA-related parental genes enriched in the GO terms. Different shapes represent different categories of GO terms. The color and size of each bubble represent the enrichment significance and the number of circRNA-related parental genes enriched in a GO term or pathway, respectively. A smaller *p*-value indicates a more significant enrichment.

GO enrichment analysis of circRNAs’ parental genes revealed significant insights into the biological processes affected by RABV infection. In mouse brain infected with the attenuated strain SRV9 (Figure S4A-C), key enriched GO terms included “synapse organization” (GO:0050808), “dendrite morphogenesis” (GO:0048813), and “axonogenesis” (GO:0007409). Cellular component analysis highlighted enrichment in “postsynaptic membrane” (GO:0045211) and “synaptic membrane” (GO:0097060). In mouse brain infected with the virulent strain CVS-11 infections (Figure S5A-C) showed differential enrichment in processes such as “dendrite development” (GO:0016358) and “regulation of synapse assembly” (GO:0051963). In the direct comparison between mice infected with SRV9 and CVS-11 strains (Figure 2A–C), enriched terms included “synapse organization” (GO:0050808), “dendrite development” (GO:0016358), and “apoptotic process” (GO:0006915). These findings suggested that RABV infection, particularly with the virulent CVS-11 strain, profoundly disrupted the molecular structure required for neuronal connectivity and synaptic function. Given that the maintenance of synaptic integrity is paramount for neuronal survival, the dysregulation of these circRNAs likely serves as a prelude to the neuronal damage and eventual cell death characteristic of RABV neuropathogenesis.

### KEGG enrichment of parental genes of circRNAs

**Figure 3.**
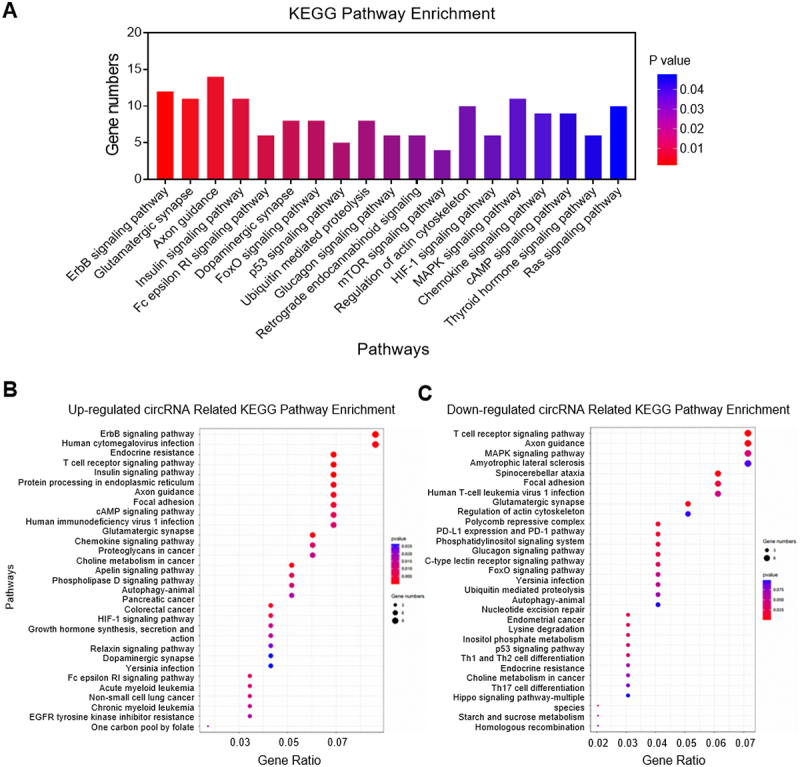
KEGG pathway enrichment analysis of differentially expressed circRNAs related parental genes in mouse brain infected with attenuated strain SRV9 compared with virulent strain CVS-11. (A) Bar plots provide an overview of the distribution of KEGG pathways among the parental genes of differentially expressed circRNAs in the comparison between SRV9 and CVS-11 group. The Y-axis represents the pathways, while the X-axis represents the number of circRNA-related parental genes enriched in each pathway. The significance level, measured by the *p*-value, is indicated by different colors. Bubble plots present the top 50 enriched KEGG pathways that were up-regulated (B) or down-regulated (C) among the parental genes of differentially expressed circRNAs in the comparison between SRV9 and CVS-11 group. The Y-axis represents the pathways, and each bubble represents a specific pathway. The color of the bubble reflects the significance level measured by the *p*-value, while the size of the bubble corresponds to the number of genes enriched in that pathway.

KEGG pathway analysis provided further insights into the molecular mechanisms affected by RABV infection. For SRV9 infection (Figure S6A-C), top enriched pathways included “Morphine addiction” (mmu05032) and “Purine metabolism” (mmu00230). In CVS-11 infection (Figure S7A-C), significant pathways included “Ubiquitin mediated proteolysis” (mmu04120) and “Endocytosis” (mmu04144). Comparing SRV9 and CVS-11 infections (Figure 3A–C) revealed differential enrichment in pathways such as “ErbB signaling pathway” (mmu04012), “Axon guidance” (mmu04360), and the “MAPK signaling pathway” (mmu04010), both of which are pivotal for maintaining neuronal cytoskeletal integrity, synaptic plasticity, and growth cone dynamics, strongly suggests a mechanistic link to the disruption of neuronal homeostasis.

### Construction and characterization of the mmu_circ_0012122 overexpression vector

**Figure 4.**
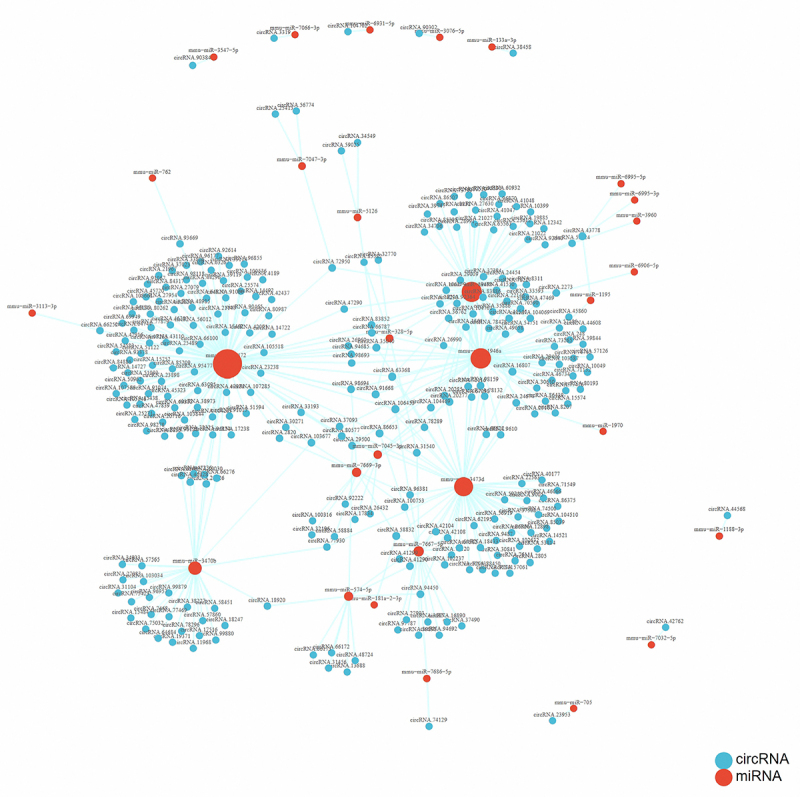
The interaction network of differentially expressed circRNAs and miRNAs in mouse brain infected with rabies virus. Red nodes represent miRNAs, and blue nodes represent circRNAs. The top 400 sponge sites are depicted in the network.

A total of 1,911 target miRNAs sponged by 1,306 DECs were predicted, with 563,113 possible binding sites. The same circRNA might have different binding sites for different miRNAs, and multiple binding sites for the same miRNA; meanwhile, different circRNAs might also have binding sites for the same miRNA. The interaction network of the top 400 binding sites between miRNAs and DECs (Figure 4). Meanwhile, we instead identified circRNAs that were consistently upregulated in both virus-infected groups compared to the control, providing a set of core host response factors to RABV infection. This intersection yielded 55 candidate unknown circRNAs, of which the top 30 are listed in the Table S2. Based on a sequential filtering process that prioritized the significance of dysregulation, expression abundance, predicted functional relevance as a strong miRNA sponge and novelty, circRNAs mmu_circ_0012122 (812 nt) was elaborately selected. Moreover, the circular structure of mmu_circ_0012122 was confirmed (Figure S8 and Table S3).

**Figure 5.**
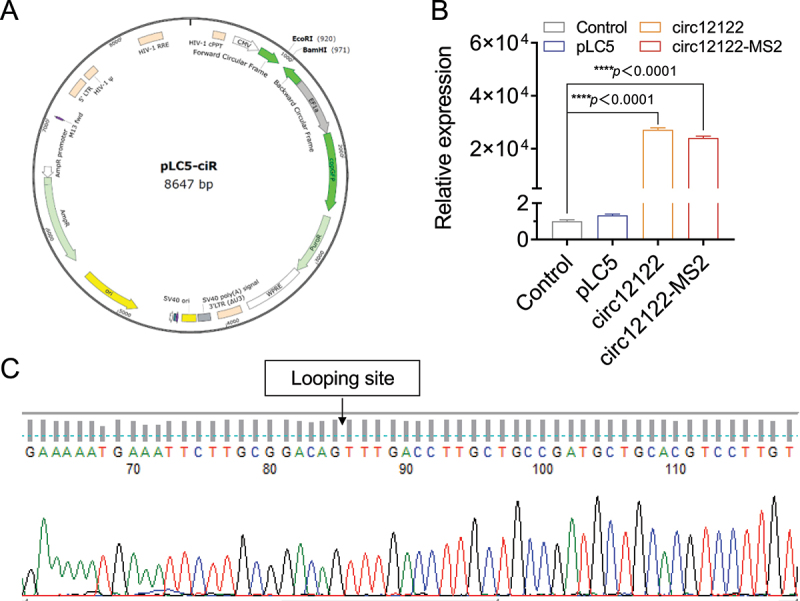
Construction and characterization of the mmu_circ_0012122 overexpression vector. (A) Overexpression vector for mmu_circ_0012122 (circ12122). (B) The relative expression of target genes in N2A cells transfected with different vectors by RT-qPCR. The control group represents untransfected N2A cells. Data are presented as means ± SD (*n* = 3). (C) Diagram of mmu_circ_0012122 looping sites. Statistical significance was determined using one-way ANOVA with Tukey’s post hoc test. ****p < 0.0001.

To further explore the regulatory mechanism of mmu_circ_0012122 and its associated miRNAs in RABV infection, overexpression vectors for mmu_circ_0012122 (circ12122) and mmu_circ_0012122 with MS2 tag (circ12122-MS2) were first constructed using In-Fusion cloning technology (Figure 5A). These vectors were then transfected into Neuro-2A (N2A) mouse neuroblastoma cells to evaluate the expression level of the target gene. The result showed that the expression level of target genes in cells transfected with circ12122 and circ12122-MS2 vectors was increased 27,167-fold and 24,069-fold, respectively, compared with the control group (Figure 5B). The exact looping of the expected junction sites was confirmed by PCR product sequencing, which was consistent with the circBase database (Figure 5C). These results indicated that circ12122 and circ12122-MS2 successfully overexpressed target circRNA in eukaryotic cells.

### Identification and screening of adsorbed miRNAs from mmu_circ_0012122

**Figure 6.**
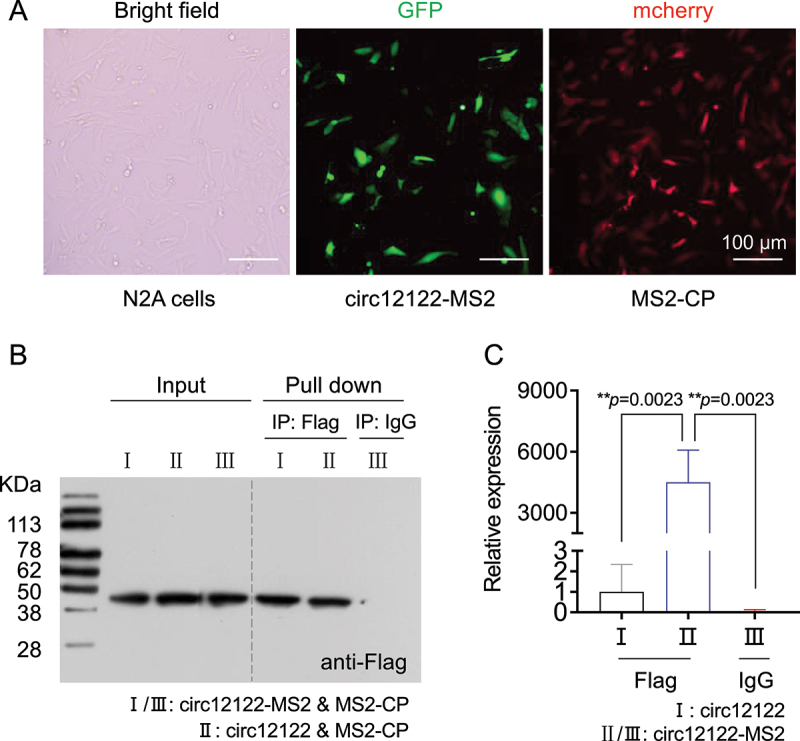
Identification of adsorbed miRNAs from mmu_circ_0012122. (A) Fluorescence images showing that circ12122-MS2 (green) and MS2-CP (red) were successfully transfected into N2A cells and expressed. Scale bar: 100 μm. (B) Western blot image showing the enrichment of MS2-CP-Flag in the pull-down products. (C) The relative expression of circ12122 and circ12122-MS2 by RT-qPCR. Data are presented as means ± SD (*n* = 3). Statistical significance was determined using one-way ANOVA with Tukey’s post hoc test. **p < 0.01.

To identify miRNAs associated with mmu_circ_0012122, an MS2-CP-Flag circRNA pull-down assay was employed. circ12122-MS2 and MS2-CP expression vectors were transfected simultaneously in N2A cells. When the MS2-CP protein was induced to express in N2A cells, it could bind specifically to circRNA with MS2 tag, thereby forming the circRNA-MS2 & MS2-CP complex. Fluorescence images showed that circ12122-MS2 (green) and MS2-CP (red) were successfully transfected into N2A cells and expressed (Figure 6A). Subsequently, the induced N2A cells were lysed and circRNA-MS2 & MS2-CP complex was pulled down. Western blot analysis demonstrated enrichment of MS2-CP-Flag in the pull-down products (Figure 6B), while RT-qPCR confirmed abundant mmu_circ_0012122 capture (Figure 6C). Subsequently, the RNA of pull-down products was extracted and sequencing was then performed using Novaseq 6000 PE150 mode. A total of 391 miRNAs were identified by pull-down product alignment of circ12122-MS2 & MS2-CP group and circ12122 & MS2-CP group, including 106 miRNAs unique to circ12122-MS2 & MS2-CP group, 12 miRNAs unique to circ12122 & MS2-CP group, and 273 miRNAs shared by both groups, which were distributed on each chromosome.

**Figure 7.**
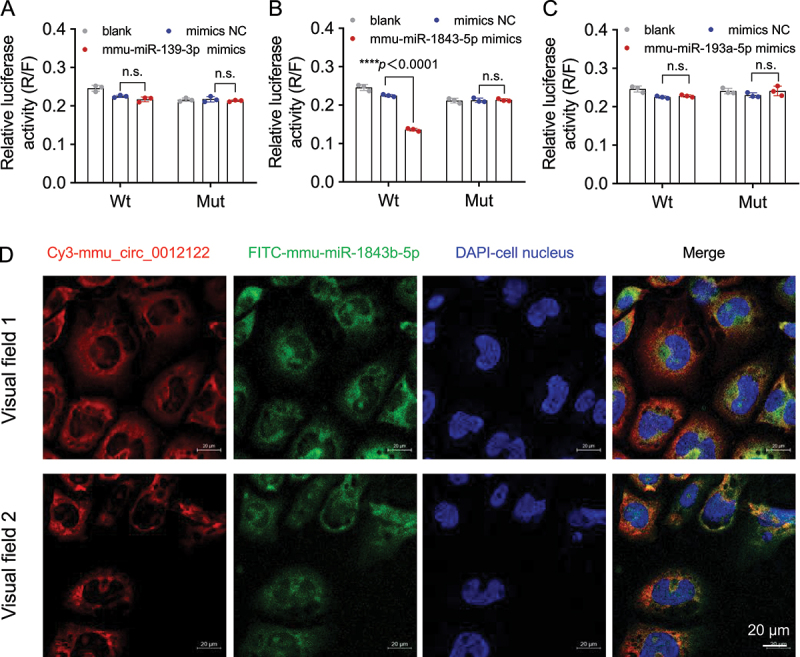
Validation of the significant interaction between mmu_circ_0012122 and mmu-miR-1843-5p. Dual-luciferase reporter assays analyzing the interaction between mmu_circ_0012122 and mmu-miR-139-3p (A), mmu-miR-1843-5p (B), or mmu-miR-193a-5p (C). Data are presented as means ± SD (*n* = 3). (D) Fluorescence in situ hybridization images showing the co-localization of mmu_circ_0012122 (red) and mmu-miR-1843-5p (blue) in the cytoplasm of N2A cells. DAPI: cell nucleus. Scale bar: 20 μm. Statistical significance was determined using one-way ANOVA with Tukey’s post hoc test. ****p < 0.0001; n.s., not significant.

Based on the intersection of high-abundance sequencing reads, strong predicted binding affinity to the circRNA sequence, and documented relevance to neuronal biology, we prioritized six candidates for experimental validation in two batches. The first batch (mmu-miR-7020-5p, mmu-miR-6975-5p, mmu-miR-7226-5p) showed no significant interaction in dual-luciferase assays, leading us to test the second batch (mmu-miR-139-3p, mmu-miR-1843-5p, mmu-miR-193a-5p). The wild-type and mutant vector were separately designed and constructed (Table S4 and Figure S9). Dual-luciferase reporter assays revealed that transfection of mmu-miR-1843-5p significantly decreased the relative fluorescence activity ratio (Renilla luciferase/Firefly luciferase, R/F) of 293T cells transfected with psi-mmu_circ_0012122-wt-psiCHECK2 compared with negative control mimics NC, confirming a significant interaction between mmu_circ_0012122 and mmu-miR-1843-5p (Figure 7A–C). Fluorescence in situ hybridization (FISH) images confirmed co-localization of mmu_circ_0012122 and mmu-miR-1843-5p in the cytoplasm of N2A cells (Figure 7D and Table S5). These data indicated that mmu-miR-1843-5p was reasonable for further validation as a targeted miRNA for mmu_circ_0012122.

### Molecular validation of a novel regulatory pathway in RABV infection: mmu_circ_0012122/mmu-miR-1843-5p/Sertad2 axis

**Figure 8.**
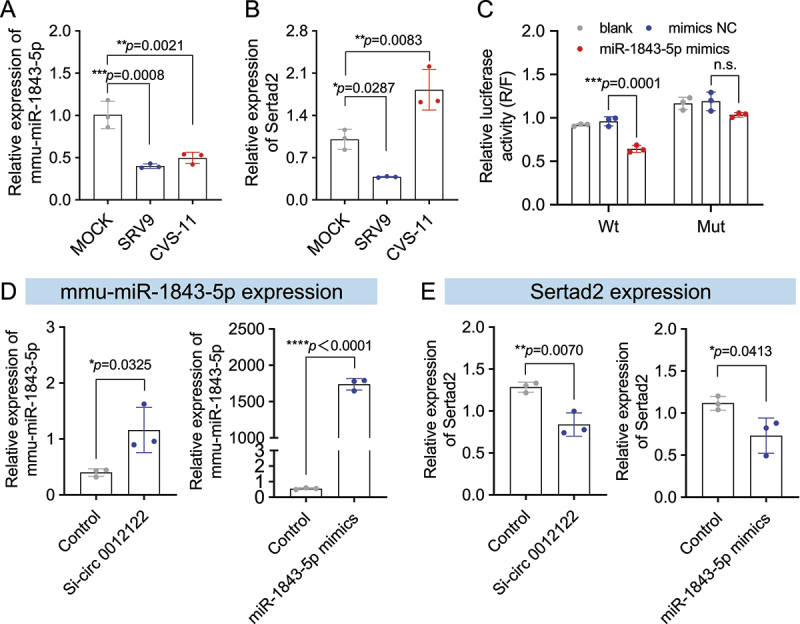
Molecular validation of a novel regulatory pathway in RABV infection: mmu_circ_0012122/mmu-miR-1843-5p/Sertad2 axis. The relative expression of mmu-miR-1843-5p (A) and Sertad2 (B) in mouse brain infected with attenuated strain SRV9 or virulent strain CVS-11 by RT-qPCR. Mice in the MOCK group were inoculated with normal mouse brain tissue suspension, namely group N as mentioned previously. Data are presented as means ± SD (*n* = 3). (C) Dual-luciferase reporter assays analysing the interaction between mmu-miR-1843-5p and Sertad2. The blank group represents untransfected 293T cells. Data are presented as means ± SD (*n* = 3). The relative expression of mmu-miR-1843-5p (D) and Sertad2 (E) in N2A cells treated with Si-circ 0012122 or mmu-miR-1843-5p mimics. The control group in (D) and (E) represents siRNAs with mismatched sequences at one end (left) or mimics NC (right). Data are presented as means ± SD (*n* = 3). Statistical significance was determined using one-way ANOVA with Tukey’s post hoc test and Student’s t-test. *p < 0.05; **p < 0.01; ***p < 0.001; ****p < 0.0001; n.s., not significant.

A total of 198 mRNA molecules were predicted through multiMiR, 16 of which have been reported in the relevant literature and verified by experiments. At the same time of circRNA sequencing in the brain tissue of mice infected with RABV, mRNA sequencing was performed on the samples at the same time, and a total of more than 30,000 differentially expressed mRNA were detected. When the detected differential mRNA intersected with 198 mRNA molecules predicted above, only one intersection molecule, Sertad2, was obtained, indicating Sertad2 as a potential target of mmu-miR-1843-5p. RT-qPCR primer sequences designed for mmu-miR-1843-5p and Sertad2 are shown in Table S6. RT-qPCR analysis of RABV-infected mouse brains showed decreased expression of mmu-miR-1843-5p and increased expression of Sertad2 in the highly virulent CVS-11 strain infection compared to controls (Figure 8A,B). A dual-luciferase reporter assay showed that transfection of mmu-miR-1843-5p significantly decreased the relative fluorescence activity ratio (R/F) of 293T cells transfected with Sertad2-3’UTR-wt-psiCHECK2 compared with negative control mimics NC, confirming a significant interaction between mmu-miR-1843-5p and the Sertad2-3’UTR (Figure 8C).

To further investigate the sponge function of mmu_circ_0012122, siRNA was designed to knockdown the expression of mmu_circ_0012122 (Si-circ 0012122) and those with mismatched sequences at one end were designed as controls. RT-qPCR experiments demonstrated that knockdown of mmu_circ_0012122 resulted in increased expression of mmu-miR-1843-5p and decreased expression of Sertad2 in N2A cells (Figure 8D,E). Conversely, overexpression of mmu_circ_0012122 increased Sertad2 levels, while mmu-miR-1843-5p overexpression decreased Sertad2 levels (Figure 8D,E). These data demonstrate that mmu_circ_0012122 functions as a miRNA sponge, regulating gene expression by sequestering its target miRNA. Our findings support a regulatory axis involving mmu_circ_0012122, mmu-miR-1843-5p, and Sertad2, consistent with the ceRNA hypothesis.

### Functional analysis of mmu_circ_0012122/mmu-miR-1843-5p/Sertad2 in nerve cell apoptosis and inflammation

**Figure 9.**
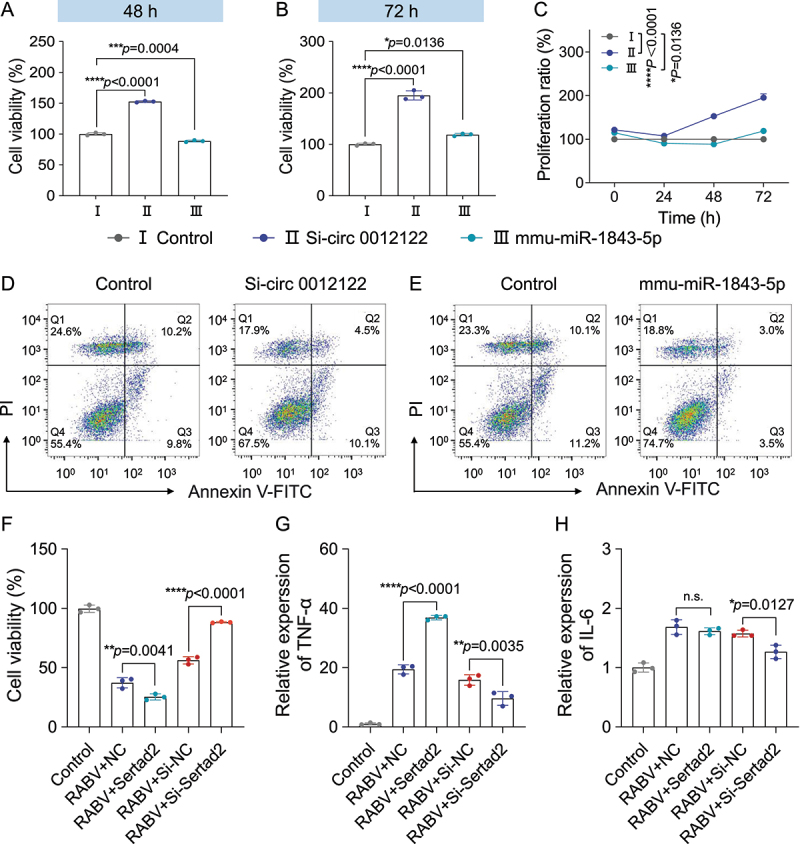
Functional analysis of mmu_circ_0012122/mmu-miR-1843-5p/Sertad2 in nerve cell apoptosis and inflammation. Cell viability of N2A cells transfected with different plasmids at 48 h (A) and 72 h (B). Data are presented as means ±SD (*n* = 3). (C) Proliferation rate of N2A cells transfected with different plasmids within 72 h. The control group in (A), (B), and (C) represents untransfected N2A cells. Data are presented as means ± SD (*n* = 3). Representative scatter plots of flow cytometry data showing the proportion of Annexin V^−^PI^+^ necrotic cell (Gate Q1), Annexin V^+^PI^+^ non-viable apoptotic cell (Gate Q2), Annexin V^+^PI^−^ viable apoptotic cells (Gate Q3) or Annexin V^−^PI^−^ live cells (Gate Q4) after treatment with Si-circ 0012122 (D) or mmu-miR-1843-5p mimics (E). The control group in (D) and (E) represents siRNAs with mismatched sequences at one end (left) or mimics NC (right). (F) quantitative analysis of non-apoptotic cells from TUNEL staining images of N2A cells under different treatments. Data are presented as means ± SD (*n* = 3). Relative expression level of TNF-α (G) and IL-6 (H) in N2A cells measured by RT-qPCR. The control group in (F), (G), and (H) represents untreated N2A cells. Statistical significance was determined using one-way ANOVA with Tukey’s post hoc test and student’s t-test. *p < 0.05; **p < 0.01; ***p < 0.001; ****p < 0.0001.

To establish a mechanistic connection with the aforementioned KEGG and GO enrichment findings, which highlighted the dysregulation of neurodevelopmental and pro-apoptotic pathways, the effect of mmu_circ_0012122/mmu-miR-1843-5p/Sertad2 on regulating nerve cell apoptosis and inflammation was further explored. CCK-8 assays demonstrated that mmu_circ_0012122 knockdown using Si-circ 0012122 significantly enhanced cell viability at 48 h, whereas mmu-miR-1843-5p overexpression initially reduced viability but showed recovery by 72 h (Figure 9A–C). Flow cytometry analysis showed mmu_circ_0012122 knockdown predominantly reduced the proportion of Annexin V^−^PI^+^ necrotic cells (Gate Q1) and Annexin V^+^PI^+^ non-viable apoptotic N2A cells (Gate Q2). Notably, the proportion of Annexin V^+^PI^−^ viable apoptotic cells (Gate Q3) was largely unaffected by mmu_circ_0012122 knockdown (Figure 9D). For the mmu-miR-1843-5p overexpression group, the proportions of Annexin V^+^PI^−^ viable apoptotic cells (Gate Q3), Annexin V^−^PI^+^ necrotic cells (Gate Q1), and Annexin V^+^PI^+^ non-viable apoptotic N2A cells (Gate Q2) were all reduced compared to the control group (Figure 9E). Taken together, these data suggested that mmu_circ_0012122 exerted a greater influence on non-viable apoptotic (Gate Q2), while mmu-miR-1843-5p overexpression primarily affects viable apoptotic cells (Gate Q3), indicating distinct yet complementary roles in regulating neuronal cell death pathways.

To further explore the function of Sertad2 in modulating neuronal responses to viral infection, Sertad2, the overexpression plasmid (Sertad2-pCDH-CMV-MCS-EF1a-GFP-puro) and siRNA (Si-Sertad2) were constructed and transfected into N2A cells, which were subsequently inoculated with RABV strain (CVS-11). TUNEL staining revealed that Sertad2 overexpression significantly increased apoptosis in infected N2A cells, while its knockdown conferred a protective effect, enhancing cell viability (Figure S10 and Figure 9F). Meanwhile, RT-qPCR analysis revealed that RABV infection markedly upregulated the expression of the pro-inflammatory cytokines TNF-α and IL-6 compared to the control group. Interestingly, Sertad2 overexpression further enhanced TNF-α expression, though it did not significantly alter IL-6 levels. In contrast, Sertad2 knockdown observably suppressed the expression of both TNF-α and IL-6, indicating a substantial attenuation of RABV-triggered neuroinflammatory responses (Figure 9G,H). These results collectively demonstrated that Sertad2 not only promoted neuronal apoptosis but also modulated neuroinflammation upon RABV infection, underscoring its functional significance in viral pathogenesis.

## Discussion

Previous studies have reported numerous conserved circRNAs in mouse and human brains [42–46], highlighting their abundance and neural enrichment. However, the role of circRNAs in RABV infection remains poorly understood. Our study revealed strain-specific circRNA dysregulation, with virulent CVS-11 inducing more DECs than attenuated SRV9, suggesting a link between circRNA profiles and viral pathogenicity. The molecular mechanisms underlying these strain-specific differences represent an important area for future investigation. our previous proteomic work provides relevant insights into potential mechanisms [28,29]. We have shown that RABV infection significantly alters the expression of key RNA-binding proteins (RBPs), including hnRNP L, which participates in splicing regulation [29]. This suggests that viral infection may modulate the host’s RNA processing machinery, potentially through interactions between viral proteins (such as P or M proteins) and host RBPs, thereby influencing circRNA biogenesis. The differential virulence of CVS-11 and SRV9 strains may stem from their distinct capabilities to manipulate these cellular pathways.

More significantly, our findings resonate with circRNA studies in other neurotropic viruses. For instance, in Zika virus (ZIKV) infection, knockdown of hsa_circ_0007321 inhibited ZIKV replication through the miR-492/NFKBID axis and activation of the NF-κB signaling pathway [47], similar to our observed mmu_circ_0012122-mediated axis. Even SARS-CoV-2, which can manifest neurological complications, has been associated with altered circRNA profiles that may influence inflammatory pathways in the central nervous system [48]. This convergence of findings across diverse viral models underscores the fundamental role of circRNA-associated ceRNA networks in virus-neuron interactions. Our work extends this paradigm to RABV infection, providing a mechanistic link between a specific circRNA-mediated axis and the regulation of neuronal survival.

Further validation in vivo revealed a distinct pattern of regulatory molecule expression. Consistent with our proposed ceRNA mechanism, CVS-11 infection led to a significant downregulation of mmu-miR-1843-5p accompanied by an upregulation of Sertad2 in mouse brains, validating the functional interaction where mmu_circ_0012122 sponges mmu-miR-1843-5p to derepress Sertad2. Interestingly, SRV9 infection presented a contrasting scenario where Sertad2 expression was lower than in MOCK controls despite the downregulation of mmu-miR-1843-5p. This suggests that the attenuated SRV9 strain may recruit additional strain-specific regulatory mechanisms, such as alternative transcriptional repressors or competing endogenous RNA networks, which complicate the linear interpretation of the circRNA-miRNA-mRNA axis in the context of attenuated infection.

The potential conservation of this regulatory axis across species enhances its biological significance. The parent gene of mmu_circ_0012122 and the Sertad2 gene are well-conserved in humans. While the specific miRNA mmu-miR-1843-5p is not highly conserved, the underlying principle of a non-coding RNA (e.g. circRNA) sponging a miRNA to regulate a key apoptotic gene like Sertad2 may be a conserved regulatory module. Future studies should investigate the existence of a homologous circuit in human neuronal models and explore its relevance to other neurological disorders. From a translational perspective, the clear functional impact of this axis on nerve cell viability highlights its promise as a therapeutic target. Targeting this circuit, for instance by inhibiting the pro-apoptotic circRNA using antisense strategies or modulating the miRNA activity, could represent a novel neuroprotective strategy to mitigate the devastating effects of RABV infection, a direction worthy of further exploration.

## Conclusion

In summary, this study delineated a novel regulatory axis, mmu_circ_0012122/mmu-miR-1843-5p/Sertad2, in RABV-infected mouse brains. Transcriptomic profiling demonstrated strain-specific circRNA dysregulation, with virulent CVS-11 infection inducing more pronounced alterations than attenuated SRV9, suggesting a link between viral pathogenicity and host circRNA expression patterns. Functional validation confirmed that mmu_circ_0012122 acts as a sponge for mmu-miR-1843-5p, thereby derepressing Sertad2 expression. Crucially, this axis differentially modulated nerve cell viability: mmu_circ_0012122 knockdown reduced necrosis and non-viable apoptosis (Annexin V^+^PI^+^), while mmu-miR-1843-5p overexpression suppressed viable apoptosis (Annexin V^+^PI^−^) and necrosis. Comprehensive characterization further identified Sertad2 as a key mediator promoting both nerve cell apoptosis and neuroinflammatory responses during infection. These findings underscored circRNAs as critical arbiters of neuronal fate during RABV infection and highlighted the mmu_circ_0012122-driven network as a potential therapeutic target. Future studies should explore this axis in human neuronal models and assess its conservation across neurotropic viruses.

## Supplementary Material

Clean Copy of Supplementary Tables - QVIR-2025-0590.R2.docx

Clean Copy of Supplementary Material - QVIR-2025-0590.R2.docx

## Data Availability

The raw RNA sequencing data that support the findings of this study are openly available in National Center for Biotechnology Information (NCBI), at https://www.ncbi.nlm.nih.gov/sra/?term=PRJNA1053907^42^, and the raw experimental data are openly available in Figshare database at https://doi.org/10.6084/m9.figshare.29939639 [49].

## Other statement

We have adhered to ARRIVE guidelines and and upload a completed checklist.
