# Supplementary material for: mmu_circ_0012122/mmu-miR-1843-5p/Sertad2 axis: A novel regulatory pathway in rabies virus infection
Source: Virulence. 2026 Jun 17;17(1):2690777. doi: 10.1080/21505594.2026.2690777 (PMC13285605; doi:10.1080/21505594.2026.2690777)
Supplement: Clean Copy of Supplementary Tables - QVIR-2025-0590.R2.docx [file KVIR_A_2690777_SM9998.docx]

**Supplementary Materials——Tables**

**mmu_circ_0012122/mmu-miR-1843-5p/Sertad2** **Axis: A Novel Regulatory Pathway in Rabies Virus Infection**

Qianni Ye^1#^, Xinggang Tang^1#^, Haiming Cai^1^, Minggui Yuan^1^, Xiaomin Ba^1^, Ya Tian^1^, Jing Chen^1^, Xiaohu Wang^1*^, Rong Xiang^1*^

^1^ Institute of Animal Health, Guangdong Academy of Agricultural Sciences; Guangdong Province Key Laboratory of Livestock Disease Prevention

^#^Equal contribution

*Corresponding authors:

Xiaohu Wang, PhD, Research Fellow, Email: [wangxiaohu@gdaas.cn](mailto:wangxiaohu@gdaas.cn)

Rong Xiang, PhD, Research Fellow, Email: [xiangrong@gdaas.cn](mailto:xiangrong@gdaas.cn;)

Institute of Animal Health, Guangdong Academy of Agricultural Sciences, Guangzhou 510640, P. R. China

**KEYWORDS**

Rabies virus, CircRNA, Expression profile, circRNA-miRNA network

Table S1. Summary of circRNA sequencing results

| Sample ID | Raw reads | Clean reads | Mapped reads | Mapped Pair Reads | Mapped broken-pair reads | Mapping ratio |
| --- | --- | --- | --- | --- | --- | --- |
| C-1 | 81,617,862 | 71,927,616 | 71,715,082 | 71,577,240 | 137,842 | 99.70% |
| C-2 | 61,449,894 | 54,198,248 | 53,949,580 | 53,803,348 | 146,232 | 99.54% |
| C-3 | 86,210,200 | 75,829,882 | 75,613,935 | 75,468,392 | 145,543 | 99.72% |
| S-1 | 87,502,904 | 76,865,704 | 76,577,669 | 76,398,144 | 179,525 | 99.63% |
| S-2 | 71,796,542 | 62,920,602 | 62,591,770 | 62,392,510 | 199,260 | 99.48% |
| S-3 | 87,798,492 | 77,944,268 | 77,584,687 | 77,364,672 | 220,015 | 99.54% |
| N-1 | 90,694,518 | 80,356,660 | 80,008,499 | 79,794,148 | 214,351 | 99.57% |
| N-2 | 88,105,544 | 77,738,862 | 77,497,691 | 77,341,744 | 155,947 | 99.69% |
| N-3 | 91,404,188 | 80,234,070 | 79,864,808 | 79,640,658 | 224,150 | 99.54% |

Table S2. Top 30 circRNAs that are the most abundant and differentially expressed across virus-infected groups

|  | circRNA ID | Type | N | S | P  value | Q  value | updown | N | C | P  value | Q  value | updown |
| --- | --- | --- | --- | --- | --- | --- | --- | --- | --- | --- | --- | --- |
| 1 | circRNA.106361 | exon | 34.9 | 417.6 | 0.0023 | 1 | UP | 34.9 | 393.2 | 0.0004 | 1 | UP |
| 2 | circRNA.106360 | exon | 8.6 | 92.0 | 0.0013 | 1 | UP | 8.6 | 77.0 | 0.0006 | 1 | UP |
| 3 | circRNA.106364 | exon | 2.1 | 30.7 | 0.0088 | 1 | UP | 2.1 | 23.9 | 0.0048 | 1 | UP |
| 4 | circRNA.48514 | exon | 2.0 | 6.4 | 0.0276 | 1 | UP | 2.0 | 5.5 | 0.0350 | 1 | UP |
| 5 | circRNA.43589 | intron | 0.0 | 5.5 | 0.0002 | 1 | UP | 0.0 | 5.2 | 0.0002 | 1 | UP |
| 6 | circRNA.8391 | exon | 0.5 | 3.4 | 0.0253 | 1 | UP | 0.5 | 3.6 | 0.0069 | 1 | UP |
| 7 | circRNA.35698 | exon | 0.8 | 2.8 | 0.0353 | 1 | UP | 0.8 | 2.9 | 0.0144 | 1 | UP |
| 8 | circRNA.75032 | exon | 0.6 | 3.3 | 0.0099 | 1 | UP | 0.6 | 2.7 | 0.0146 | 1 | UP |
| 9 | circRNA.31081 | exon | 0.3 | 2.2 | 0.0116 | 1 | UP | 0.3 | 2.5 | 0.0035 | 1 | UP |
| 10 | circRNA.42181 | exon | 0.2 | 2.6 | 0.0223 | 1 | UP | 0.2 | 2.5 | 0.0152 | 1 | UP |
| 11 | circRNA.46771 | exon | 0.3 | 2.4 | 0.0290 | 1 | UP | 0.3 | 2.4 | 0.0163 | 1 | UP |
| 12 | circRNA.26960 | exon | 0.1 | 3.0 | 0.0010 | 1 | UP | 0.1 | 2.3 | 0.0245 | 1 | UP |
| 13 | circRNA.15462 | intergenic_region | 0.1 | 2.6 | 0.0007 | 1 | UP | 0.1 | 1.8 | 0.0014 | 1 | UP |
| 14 | circRNA.21767 | exon | 0.0 | 1.0 | 0.0187 | 1 | UP | 0.0 | 1.7 | 0.0042 | 1 | UP |
| 15 | circRNA.105724 | exon | 0.2 | 1.9 | 0.0311 | 1 | UP | 0.2 | 1.7 | 0.0326 | 1 | UP |
| 16 | circRNA.83666 | exon | 0.1 | 1.6 | 0.0236 | 1 | UP | 0.1 | 1.6 | 0.0132 | 1 | UP |
| 17 | circRNA.9507 | exon | 0.0 | 0.7 | 0.0452 | 1 | UP | 0.0 | 1.6 | 0.0002 | 1 | UP |
| 18 | circRNA.62020 | exon | 0.0 | 0.9 | 0.0166 | 1 | UP | 0.0 | 1.6 | 0.0004 | 1 | UP |
| 19 | circRNA.14338 | exon | 0.0 | 1.3 | 0.0023 | 1 | UP | 0.0 | 1.5 | 0.0003 | 1 | UP |
| 20 | circRNA.33620 | exon | 0.0 | 0.9 | 0.0140 | 1 | UP | 0.0 | 1.5 | 0.0007 | 1 | UP |
| 21 | circRNA.23898 | exon | 0.0 | 1.4 | 0.0036 | 1 | UP | 0.0 | 1.5 | 0.0005 | 1 | UP |
| 22 | circRNA.48622 | exon | 0.0 | 1.3 | 0.0045 | 1 | UP | 0.0 | 1.4 | 0.0014 | 1 | UP |
| 23 | circRNA.38306 | exon | 0.0 | 2.7 | 0.0017 | 1 | UP | 0.0 | 1.4 | 0.0116 | 1 | UP |
| 24 | circRNA.59350 | exon | 0.0 | 1.5 | 0.0009 | 1 | UP | 0.0 | 1.4 | 0.0008 | 1 | UP |
| 25 | circRNA.48985 | exon | 0.0 | 1.5 | 0.0009 | 1 | UP | 0.0 | 1.3 | 0.0009 | 1 | UP |
| 26 | circRNA.20151 | exon | 0.1 | 1.4 | 0.0388 | 1 | UP | 0.1 | 1.3 | 0.0454 | 1 | UP |
| 27 | circRNA.97787 | exon | 0.0 | 0.9 | 0.0207 | 1 | UP | 0.0 | 1.3 | 0.0021 | 1 | UP |
| 28 | circRNA.41299 | exon | 0.0 | 1.1 | 0.0278 | 1 | UP | 0.0 | 1.2 | 0.0020 | 1 | UP |
| 29 | circRNA.51366 | intergenic_region | 0.0 | 1.0 | 0.0339 | 1 | UP | 0.0 | 1.2 | 0.0160 | 1 | UP |
| 30 | circRNA.103844 | exon | 0.0 | 1.4 | 0.0127 | 1 | UP | 0.0 | 1.2 | 0.0203 | 1 | UP |

Table S3. Primers used for validating the circRNAs

| circRNAs | primer | Oligonucleotide Sequences(5’-3’) |
| --- | --- | --- |
| mmu_circ_0012122 | Divergent primer | TTCTTGCGGACAGTTTGACC |
|  |  | GCCCTGTTCCTGAATCAGAC |
|  | Convergent primer | GACAAGCCTGGGAGAAGAGT |
|  |  | TCGCTCCGTTTCAGCTAGG |

Table S4. Vector sequence

| Vector | Sequences |
| --- | --- |
| psi-mmu_circ_0012122-812bp-wt-psiCHECK2 (wild type) | tttgaccttgctgccgatgctgcacgtccttgtgccttttacatcaataagccagccgaaagcccaagttcttggttgtctgattcaggaacagggctgacttactggaaactggaggagaaggacatgtatcactctttgcctgaaactttggagaagacgtttgcaccatccccagcagagaggcccctgagccaggtcctgactcttgatccaggagccatacgcatgaagccaaaggagcatgtcgcagggatccaagcccatggctttctgcatgctcttgacgacagaatatccttttccccagactccgttctggaaccaagcctgtctcgtcactctgacactgactcgtcttcacaagcaagtcataatccttcccaggtgtctgggttctccaagtatccttcaaccacgagagcatcacctgtggacacttggaaaaaccatgcattccaaagggaaagtaggaccagctccaccatcccttcacgctacaccatcactagcaacgatatctcagtcaaaactgtagacgaagagaacactgtcacagtggcctcagtcagtcagtcccagcttccaggtacagccaacagtgtcccagaatgcatttcattggcttccctggaagatcctgtgatgttgtctaagatcaggcagaacctcaaggagaagcatgcccgacacgtggccgaccttcgtgcttattatgagtcggagataagtagtctgaaacagaaactggaggccaaagacatttctgccgttgaagagtggaagaagaaaaatgaaattcttgcggacag |
| psi-mmu-miR-139-3p vs mmu_circ_0012122-812bp-mut-psiCHECK2 (mutant type) | tttgaccttgctgccgatgctgcacgtccttgtgccttttacatcaataagccagccgaaagcccaagttcttggttgtctgattcaggaacagggctgacttactggaaactggaggagaaggacatgtatcactctttgcctgaaactttggagaagacgtttgcaccatccccagcagagaggcccctgagccaggtcctgactcttgatccaggagccatacgcatgaagccaaaggagcatgtcgcagggatccaagcccatggctttctgcatgctcttgacgacagaatatccttttccccagactccgttctggaaccaagcctgtctcgtcactGtgTGTctCaGtGCAGAtGacaagcaagtcataatccttcccaggtgtctgggttctccaagtatccttcaaccacgagagcatcacctgtggacacttggaaaaaccatgcattccaaagggaaagtaggaccagctccaccatcccttcacgctacaccatcactagcaacgatatctcagtcaaaactgtagacgaagagaacactgtcacagtggcctcagtcagtcagtcccagcttccaggtacagccaacagtgtcccagaatgcatttcattggcttccctggaagatcctgtgatgttgtctaagatcaggcagaacctcaaggagaagcatgcccgacacgtggccgaccttcgtgcttattatgagtcggagataagtagtctgaaacagaaactggaggccaaagacatttctgccgttgaagagtggaagaagaaaaatgaaattcttgcggacag |
| psi-mmu-miR-1843b-5p vs mmu_circ_0012122-812bp-mut-psiCHECK2 (mutant type) | tttgaccttgctgccgatgctgcacgtccttgtgccttttacatcaataagccagccgaaagcccaagttcttggttgtctgattcaggaacagggctgacttactggaaactggaggagaaggacatgtatcactctttgcctgaaactttggagaagacgtttgcaccatccccagcagagaggcccctgagccaggtcctgactcttgatccaggagccatacgcatgaagccaaaggagcatgtcgcagggatccaagcccatggctttctgcatgctcttgacgacagaatatccttttccccagactccgttctggaaccaagcctgtctcgtcactctgacactgactcgtcttcacaagcaagtcataatccttcccaggtgtctgggttctccaagtatccttcaaccacgagagcatcacctgtggacacttggaaaaaccatgcattccaaagggaaagtaggaccagctccaccatcccttcacgctacaccatcactagcaacgatatctcagtcaaaactgtagacgaagagaacactgtcacagtggcctcagtcagtcagtcccagcttccaggtacagccaacagtgtcccagaatgcatttcattggcttccctggaagatcctgtgatgttgtctaagaAGTCgGTCTTGGAGaTggagaagcatgcccgacacgtggccgaccttcgtgcttattatgagtcggagataagtagtctgaaacagaaactggaggccaaagacatttctgccgttgaagagtggaagaagaaaaatgaaattcttgcggacag |
| psi-mmu-miR-193a-5p vs mmu_circ_0012122-812bp-mut-psiCHECK2 (mutant type) | tttgaccttgctgccgatgctgcacgtccttgtgccttttacatcaataagccagccgaaagcccaagttcttggttgtctgattcaggaacagggctgacttactggaaactggaggagaaggacatgtatcactctttgcctgaaactttggagaagacgtttgcaccatccccagcagagaggcccctgagccaggtcctgactcttgatccaggagccatacgcatgaagccaaaggagcatgtcgcagggatccaagcccatggctttctgcatgctcttgacgacagaatatccttttccccagactccgttctggaaccaagcctgtctcgtcactctgacactgactcgtcttcacaagcaagtcataatccttcccaggtgtctgggttctccaagtatccttcaaccacgagagcatcacctgtggacacttggaaaaaccatgcattccaaagggaaagtaggaccagctccaccatcccttcacgctacaccatcactagcaacgatatctcagtcaaaactgtagacgaagagaacactgtcacagtggcctcagtcagtcagtcccagcttccaggtacagccaacagtgtcccagaatgcatttcattggGAAcGGtCgTTCTtGGtgtgatgttgtctaagatcaggcagaacctcaaggagaagcatgcccgacacgtggccgaccttcgtgcttattatgagtcggagataagtagtctgaaacagaaactggaggccaaagacatttctgccgttgaagagtggaagaagaaaaatgaaattcttgcggacag |

Table S5. Primers used for fluorescence in situ hybridization

| RNA | Primers |
| --- | --- |
| mmu_circ_0012122 | 5’ CY3-AGCAAGGTCAAACTGTCCGCAAGA - 3’ CY3 |
| mmu-miR-1843b-5p | 5’ FITC-AAGTCAGACAGAGACCTCCAT - 3’ FITC |

Table S6. Primers used for fluorescence in situ hybridization

| Genes | | Primers |
| --- | --- | --- |
| β-actin | β-actin-F | GCTTCTAGGCGGACTGTTAC |
|  | β-actin-R | CCATGCCAATGTTGTCTCTT |
| U6 | U6-F | CTCGCTTCGGCAGCACA |
|  | U6-R | AACGCTTCACGAATTTGCGT |
| Sertad2 | Sertad2-F | GCAAGCCCACCAAGAGTTA |
|  | Sertad2-R | TACTGCCAGGAAGCGTCTA |
| mmu-miR-1843b-5p | mmu-miR-1843b-5p-F | CGCGATGGAGGTCTCTGTC |
|  | mmu-miR-1843b-5p-R | AGTGCAGGGTCCGAGGTATT |
