# Supplementary material for: mmu_circ_0012122/mmu-miR-1843-5p/Sertad2 axis: A novel regulatory pathway in rabies virus infection
Source: Virulence. 2026 Jun 17;17(1):2690777. doi: 10.1080/21505594.2026.2690777 (PMC13285605; doi:10.1080/21505594.2026.2690777)
Supplement: Clean Copy of Supplementary Material - QVIR-2025-0590.R2.docx [file KVIR_A_2690777_SM9997.docx]

**Supplementary Materials——Figures**

**mmu_circ_0012122/mmu-miR-1843-5p/Sertad2** **Axis: A Novel Regulatory Pathway in Rabies Virus Infection**

Qianni Ye^1#^, Xinggang Tang^1#^, Haiming Cai^1^, Minggui Yuan^1^, Xiaomin Ba^1^, Ya Tian^1^, Jing Chen^1^, Xiaohu Wang^1*^, Rong Xiang^1*^

^1^ Institute of Animal Health, Guangdong Academy of Agricultural Sciences; Guangdong Province Key Laboratory of Livestock Disease Prevention

^#^Equal contribution

*Corresponding authors:

Xiaohu Wang, PhD, Research Fellow, Email: [wangxiaohu@gdaas.cn](mailto:wangxiaohu@gdaas.cn)

Rong Xiang, PhD, Research Fellow, Email: [xiangrong@gdaas.cn](mailto:xiangrong@gdaas.cn;)

Institute of Animal Health, Guangdong Academy of Agricultural Sciences, Guangzhou 510640, P. R. China

**KEYWORDS**

Rabies virus, CircRNA, Expression profile, circRNA-miRNA network


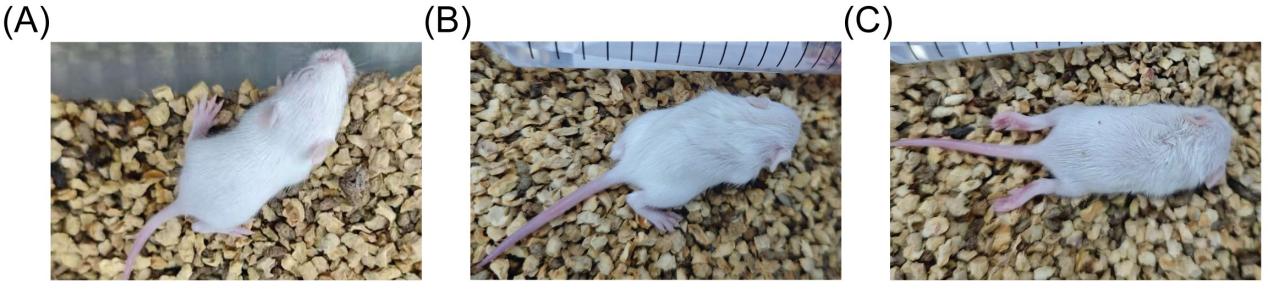


**Figure S1. Representative images of mice infected with rabies virus (RABV).** **(A)** Uninfected healthy 10-day-old mice; **(B)** Mice infected with the SRV9 strain exhibited symptoms such as trembling, weak hindlimb, and depression; **(C)** Mice infected with the CVS-11 strain showed symptoms such as ruffled fur, hindlimb paralysis and poor mental state.

**Figure S2. Genomic coverage distribution of differentially expressed circRNAs in each group.** The three comparison groups, from left to right, were SRV9 vs control, CVS-11 vs control, and SRV9 vs CVS-11. The outer circle represents the genome of mouse, with chromosome numbers marked on it; the second circle (light blue) shows the coverage distribution of circRNAs on the chromosomes. The inner circle consists of three radial lines of gray (no significant difference in expression), red (significantly upregulated circRNAs), and blue (significantly downregulated circRNAs). The length that the line extends outward represents the fold change of differentially expressed circRNAs.


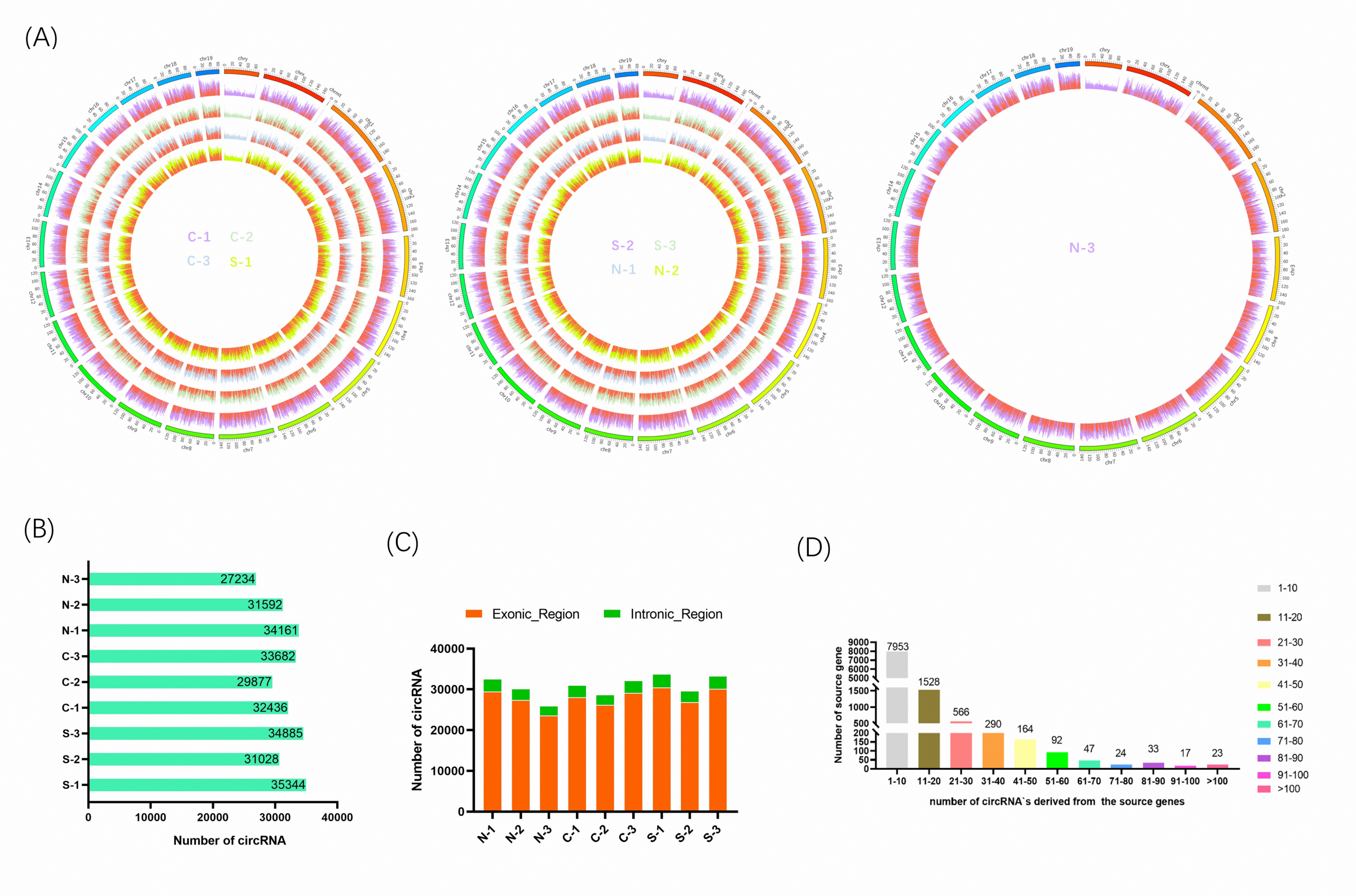


**Figure S3. Distribution of amount of circRNAs derived from different source genes.**


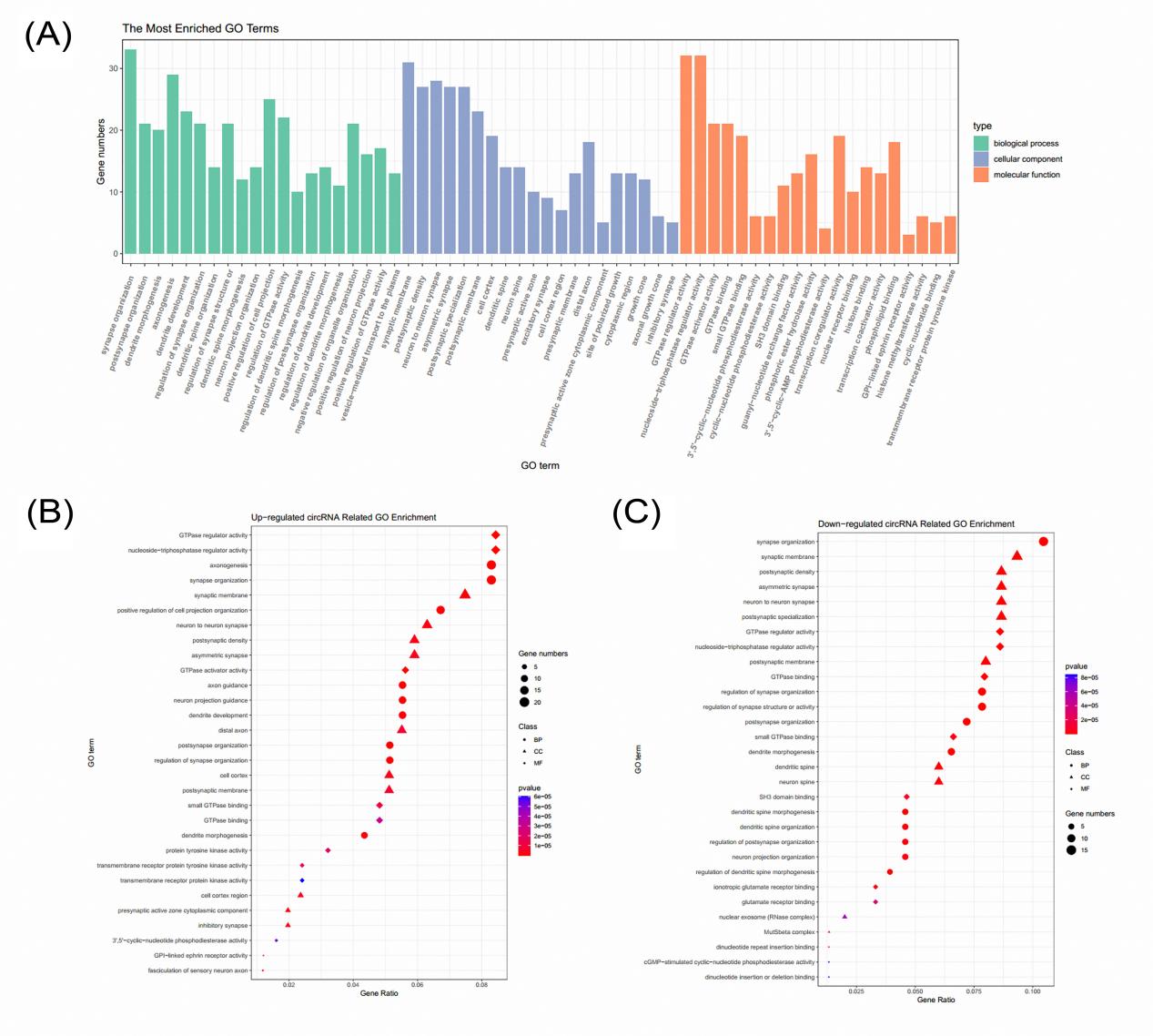


**Figure S4. GO enrichment analysis of differentially expressed circRNAs related parental genes in mouse brain infected with attenuated strain SRV9 compared with normal group.** **(A)** Bar plots illustrating the representation of GO terms in Group S vs Group N. The Y-axis represents the amount of the circRNAs related parental genes enriched in GO terms; Y-axis represents the enriched GO terms. Different colors indicate biological processes, cellular components, and molecular functions. **(B)-(C)** Scatter plots displaying the top 10 enriched GO terms for the differentially expressed circRNAs related parental genes in Group S vs Group N. Y-axis represents GO terms; X-axis represents the gene radio of the circRNAs related parental genes enriched in GO terms. Different shapes indicate different categories of GO terms: biological processes, cellular components, and molecular functions are represented by circles, squares, and triangles, respectively. The color and size of each bubble represent enrichment significance and the number of circRNAs-related parental genes enriched in a GO term or pathway, respectively. The p-value with a smaller value indicating a more significant enrichment. Figure B represents the results for up-regulated circRNAs, while Figure C represents the results for down-regulated circRNAs.


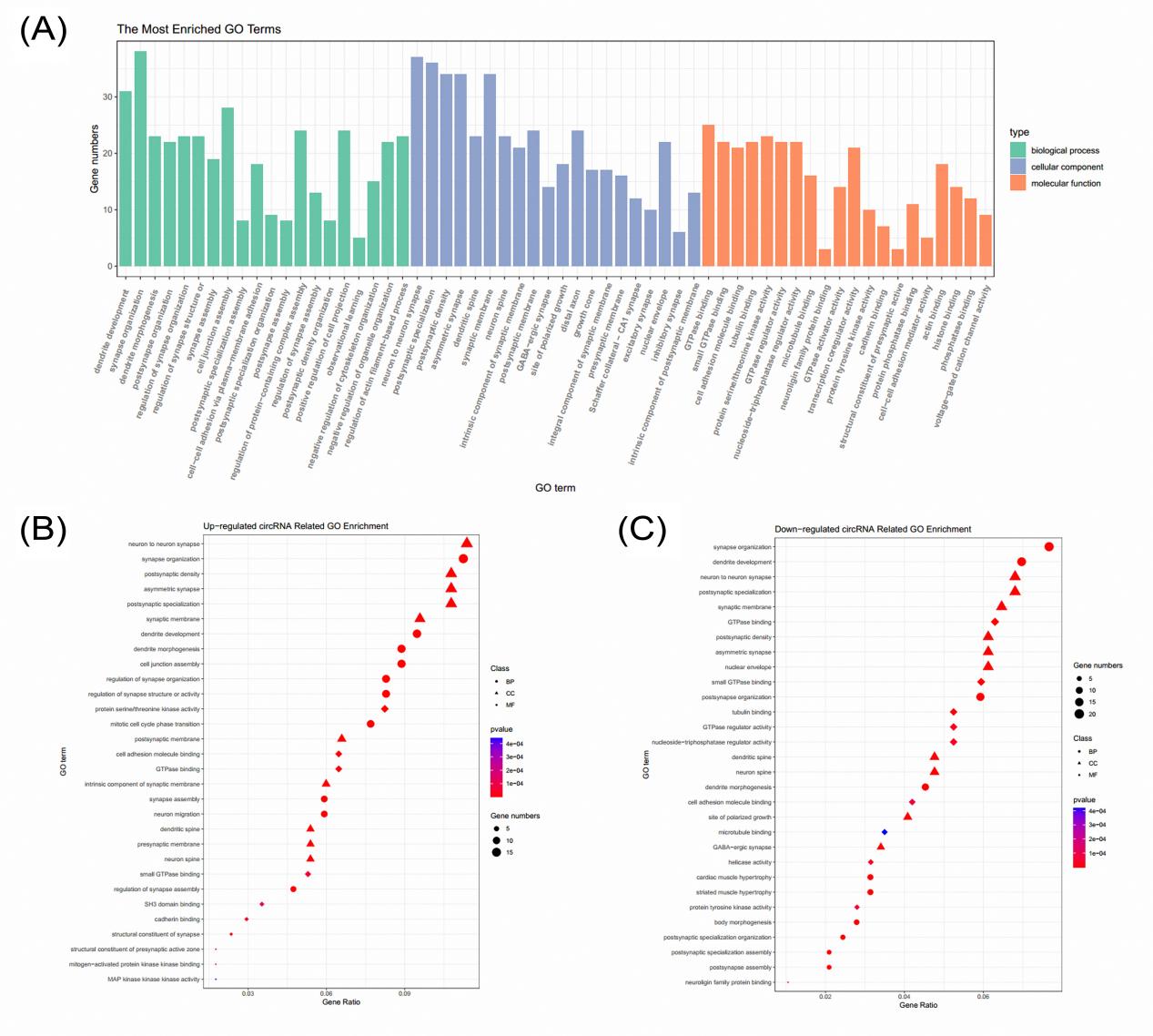


**Figure S5. GO enrichment analysis conducted on differentially expressed circRNAs and their related parental genes in normal group compared with mouse brain infected with virulent strain CVS-11. (A)** Bar plot depicts the representation of GO terms in Group N (normal group) compared to Group C (CVS-11 infected group). The Y-axis represents the number of circRNA-related parental genes enriched in GO terms, while the X-axis represents the specific GO terms. Distinct colors are used to indicate different categories, including biological processes, cellular components, and molecular functions. **(B)-(C)** Scatter plots show the top 10 enriched GO terms for differentially expressed circRNAs' related parental genes in Group N versus Group C. Figure B corresponds to the results for up-regulated circRNAs, while Figure C corresponds to the results for down-regulated circRNAs. The Y-axis represents the GO terms, while the X-axis represents the gene ratio of the circRNA-related parental genes enriched in those GO terms. Various shapes are employed to represent different GO term categories: circles for biological processes, squares for cellular components, and triangles for molecular functions. The color and size of each data point reflect the significance of enrichment and the number of circRNA-related parental genes enriched in a specific GO term or pathway, respectively. A smaller p-value indicates a more significant enrichment.


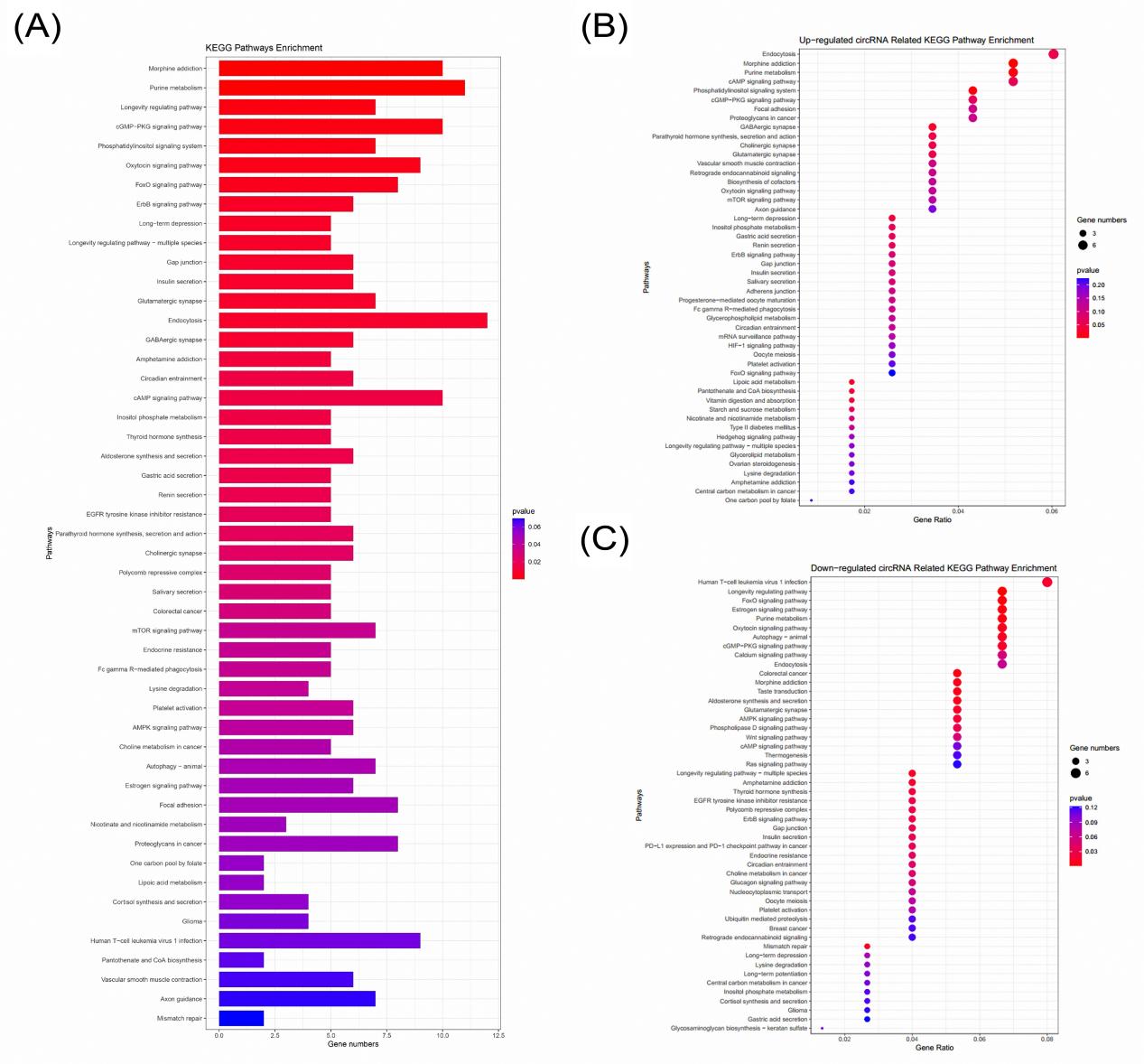


**Figure S6. KEGG pathway enrichment analysis of differentially expressed circRNAs related parental genes in mouse brain infected with attenuated strain SRV9 compared with normal group. (A)** Bar plots illustrating the distribution of KEGG pathways among the parental genes of differentially expressed circRNAs in Group S vs Group N. The Y-axis represents the pathways, while the X-axis represents the number of circRNAs related parental genes enriched in each pathway. Different colors indicate p-value. **(B)-(C)** Scatter plots displaying the top 50 enriched KEGG pathways among the parental genes of differentially expressed circRNAs in Group S vs Group N. The Y-axis represents the pathways. Each bubble in the scatter plot represents a pathway, with the color indicating the p-value and the size representing the number of genes enriched in that pathway. Figure B represents the results for up-regulated circRNAs, while Figure C represents the results for down-regulated circRNAs.


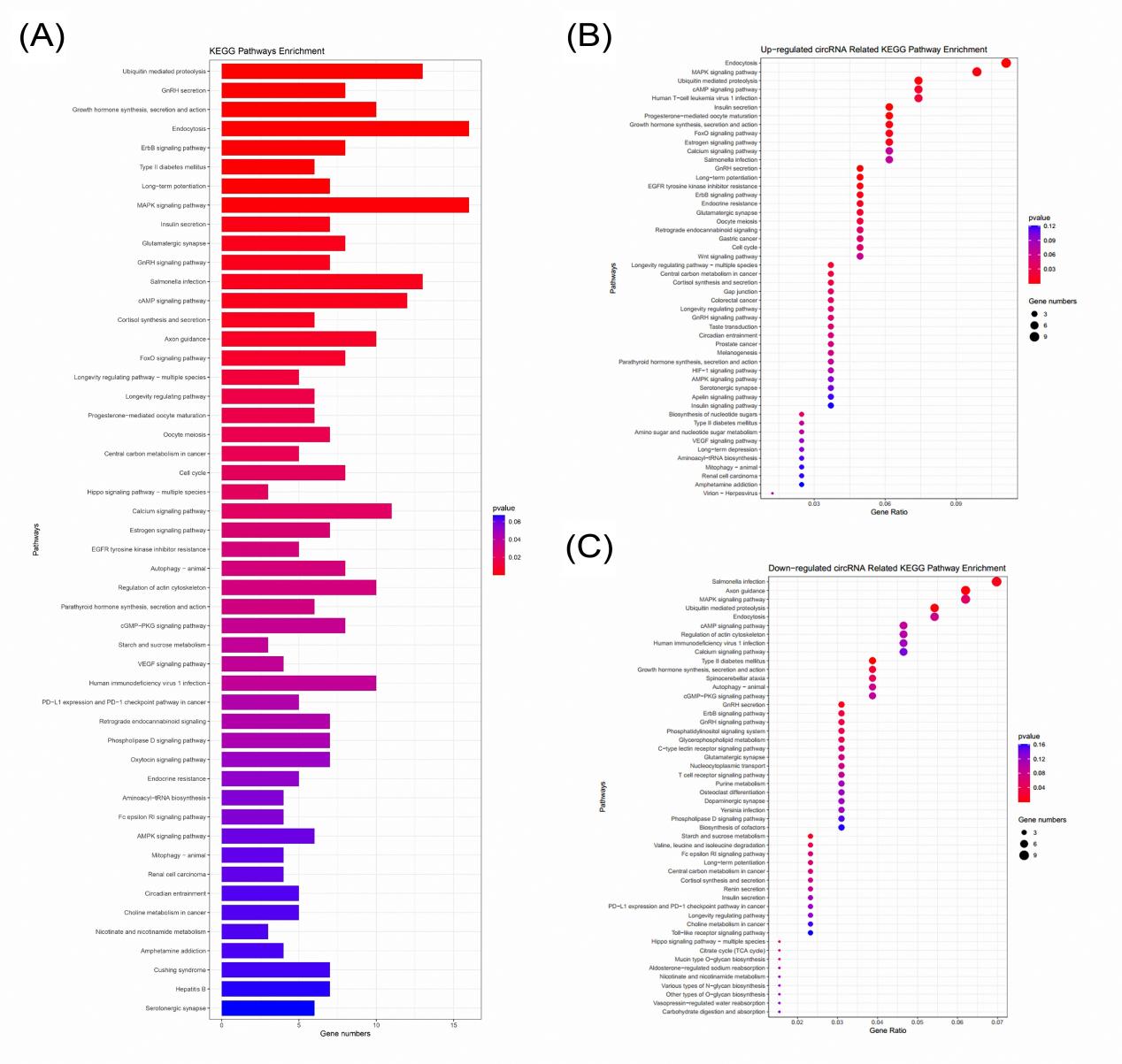


**Figure S7. KEGG pathway enrichment analysis of differentially expressed circRNAs related parental genes in normal group compared with mouse brain infected with virulent strain CVS-11. (A)** Bar plots illustrate the distribution of KEGG pathways among the parental genes of differentially expressed circRNAs in Group N vs Group C. The Y-axis represents the pathways, while the X-axis represents the number of circRNA-related parental genes enriched in each pathway. Different colors indicate the significance level measured by the p-value. **(B)-(C)** Scatter plots display the top 50 enriched KEGG pathways among the parental genes of differentially expressed circRNAs in Group N vs Group C. Figure B represents the results for up-regulated circRNAs, while Figure C represents the results for down-regulated circRNAs. The Y-axis represents the pathways, and each bubble in the scatter plot represents a pathway. The color of the bubble indicates the significance level measured by the p-value, while the size of the bubble represents the number of genes enriched in that pathway.

**
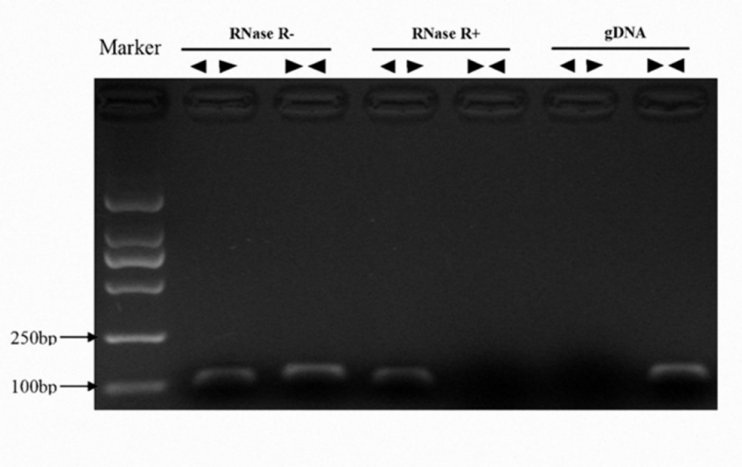
**

**Figure S8.** **Verification of circular structure for mmu_circ_0012122 using RNase R-treated and untreated RNA, as well as gDNA.** Convergent primers successfully amplified linear RNAs from both cDNA and gDNA samples. However, divergent primers exclusively amplified circRNAs from cDNA and not from gDNA. The amplified PCR products were visualized through 1% agarose gel electrophoresis.

**Figure S9. Schematic diagrams of the plasmid constructions listed in Table S4.** (A) Vector: psi-mmu_circ_0012122-812bp-wt-psiCHECK2 (wild type); (B) Vector: psi-mmu-miR-139-3p vs mmu_circ_0012122-812bp-mut-psiCHECK2 (mutant type); (C) Vector: psi-mmu-miR-1843b-5p vs mmu_circ_0012122-812bp-mut-psiCHECK2 (mutant type); (D) Vector: psi-mmu-miR-193a-5p vs mmu_circ_0012122-812bp-mut-psiCHECK2 (mutant type).


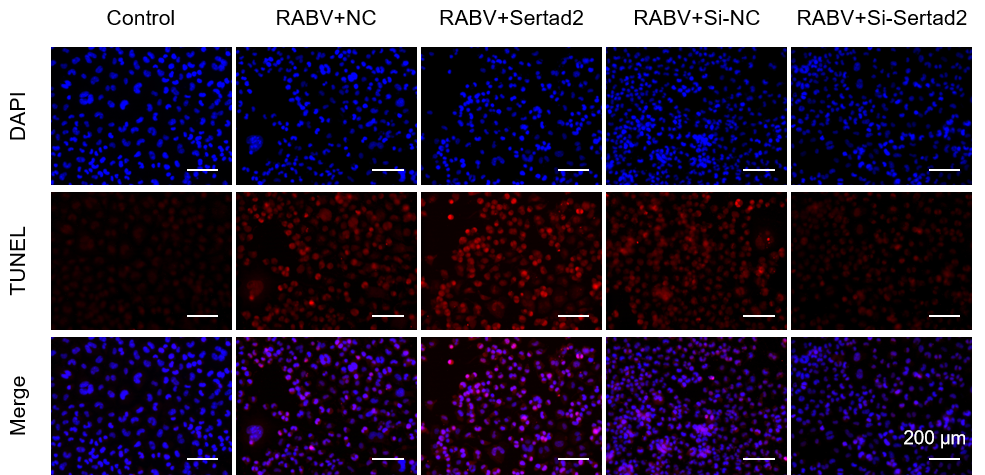


**Figure S10. Representative TUNEL staining images of N2A cells under different treatments. Red fluorescence indicates TUNEL-positive apoptotic cells. Scale bar: 200 µm.**
